# Using Serially Collected Specimens to Investigate the Potential Population Genetic Consequences of Reported Declines in Eastern Woodland Salamanders

**DOI:** 10.1002/ece3.72805

**Published:** 2026-01-18

**Authors:** Kyle A. O'Connell, Carly R. Muletz‐Wolz, Addison Wynn, Karen R. Lips, Amy Ellison, Kelly R. Zamudio, Rayna C. Bell

**Affiliations:** ^1^ Global Genome Initiative, National Museum of Natural History Smithsonian Institution Washington DC USA; ^2^ Department of Vertebrate Zoology, National Museum of Natural History Smithsonian Institution Washington DC USA; ^3^ Deloitte Consulting LLP Biomedical Data Science Team Arlington Virginia USA; ^4^ Center for Conservation Genomics, National Zoo & Conservation Biology Institute Smithsonian Institution Washington DC USA; ^5^ Department of Biology University of Maryland College Park Maryland USA; ^6^ School of Natural Sciences Bangor University Bangor UK; ^7^ Department of Integrative Biology University of Texas at Austin Austin Texas USA; ^8^ Department of Herpetology California Academy of Sciences San Francisco California USA

**Keywords:** genetic diversity, historical DNA, museum genomics, *Plethodon* salamanders, population declines, time series analysis

## Abstract

Biodiversity is facing global change at an unprecedented rate. Understanding how populations have responded to accelerated change over the last century is key to informing effective conservation policies. Serially collected specimens from natural history repositories can provide a window into how populations change over time and highlight further vulnerabilities in remaining populations. Changes in observed population abundance during field surveys suggest that some *Plethodon* salamander populations experienced declines since the 1960s, but the potential population genetic consequences of these declines remain unstudied. Thanks to decades of sustained collection‐based efforts, *Plethodon* salamanders serve as a model to test the utility of historical DNA to identify shifts in genetic diversity at recent time scales. Here, we investigate demographic change in six *Plethodon* species through time using DNA from formalin‐fixed museum specimens (1960s–1970s), historic frozen blood (1980s–1990s), and contemporary sampling. We generated several reduced representation SNP datasets using a target‐capture approach to investigate two sites in the Appalachian Range: one with documented declines (Indian Grave Gap) and one without (Skull's Gap). We quantify the impact of bioinformatic choices on estimates of genetic diversity, quantify demographic shifts, and trace changes in allele frequencies in immune‐related loci to explore the potential impact of pathogens on putative declines. We found consistent patterns of genetic diversity change across datasets and filtering regimes. At Skull's Gap, our results suggest that populations were stable or expanding, while at Indian Grave Gap, our results suggest contraction in one species and mixed signals of contraction and expansion in the others. Analyses of immune loci suggest that balancing selection is maintaining shared polymorphism through time in all but one species. Our study outlines important considerations for leveraging historical DNA in time series collections to quantify the genomic effects of localized population declines.

## Graphical abstract

Biodiversity is facing global change at an unprecedented rate; understanding how species respond to this accelerated change is important to inform future environmental and wildlife‐related policies. Serially collected specimens from natural history repositories can provide a unique window into how populations change over time and highlight further potential vulnerabilities in remaining populations. Here, we estimate levels of genetic diversity in six *Plethodon* species through time using DNA from museum specimens and contemporary sampling. We find changes in genetic diversity through time in some species and balancing selection acting on immune loci.

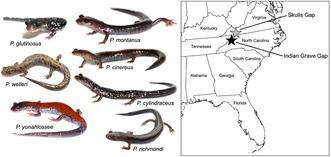

## 1 Background

Understanding how species have responded and adapted to changing environmental conditions over the last century is essential to inform future conservation efforts. Serially collected samples with specimens from a single locality across multiple time points, such as those stewarded in natural history collections, can provide a unique window into population and species‐level changes through time including overall shifts in genetic diversity and changes in allele frequencies (Habel et al. 2014). Though growing, few studies use historical DNA with serial sampling to investigate population genomic questions because techniques to reliably sequence high‐quality historical DNA datasets from museum specimens have only recently been developed (Bi et al. 2019; Byerly et al. 2024; Gauthier et al. 2020; Jensen et al. 2018; Pacioni et al. 2015; Van Der Valk et al. 2019). Historical DNA can present difficulties for population‐scale studies because historical specimens typically were not preserved for genetic analysis and thus the remaining DNA is often damaged. DNA extractions from these tissues are often of lower quality and concentration, and depending on how specimens or tissues were handled there can be high rates of exogenous contamination (Ruiz‐Gartzia et al. 2022). This lower quality starting material presents several bioinformatic challenges, including shorter high‐quality sequence reads and low amounts of endogenous DNA, which result in poorer reference mapping and de novo assembly, fewer variable sites, and a homozygous bias relative to modern samples (Cook et al. 2014; Hahn et al. 2022; O'Connell et al. 2022). Increasingly, historical genomic studies employ whole‐genome shotgun sequencing approaches resulting in partial to complete low‐coverage sequences for mitochondrial and/or nuclear genomes when a high‐quality reference genome is available (Andrews et al. 2025; Hahn et al. 2022). Unfortunately for non‐model organisms with large genomes, shotgun sequencing is often cost‐prohibitive or impractical without access to a high‐quality reference genome and thus genetic studies of these taxa require reduced representation approaches such as target capture.

Designing targets for capture in the absence of a reference genome requires either a reference transcriptome, which will produce target sequences composed primarily of exons with some flanking intron regions, or short RADseq loci, which will produce short anonymous target sequences from across the genome. Across all genomic data types (whole genomes, exons, anonymous SNPs), disentangling the effects of recent demographic declines versus biases between archival versus modern samples can be difficult. This is because post‐processing of genomic variants can strongly influence estimates of genetic diversity in historical versus contemporary populations, particularly for low‐quality sequences, potentially leading to misleading conclusions (Ewart et al. 2019; O'Connell et al. 2022). In addition, genomes carry the record of many ancient demographic processes and therefore extensive, high‐quality datasets are often necessary to differentiate between recent versus deeper evolutionary time scale events (Oaks et al. 2020). Despite these challenges, historical samples have the potential to be of great benefit to studies of species declines. In particular, collections in natural history museums contain temporal sampling that spans periods of documented declines across diverse biomes and organisms. To inform how best to leverage valuable and irreplaceable historical samples, more exploratory work is needed to understand how factors such as levels of missing data, marker choice (i.e., coding vs. non‐coding sites), and sequencing depth can impact estimates of genetic diversity and population demography through time.

One enigmatic case of species declines in North America was first proposed by Richard Highton (Highton 2005), who documented changes in salamander communities across the Appalachian mountain range between the 1950s and the 1990s. *Plethodon* salamanders are the most common vertebrates in some Appalachian ecosystems, and as such have strong top‐down regulatory effects on the trophic ecology of those ecosystems (Best and Welsh Jr. 2014; Grant et al. 2024; Hickerson et al. 2017). Based on serial survey data from 1953 until the 1990s, Highton identified synchronous population declines dating to the early 1980s in 180 of 205 populations spanning 38 *Plethodon* species, including one community at a site called Indian Grave Gap in southern Appalachia. Highton hypothesized that declines across Appalachia were driven by deforestation, climate change, or acid rain but did not find strong support for a single hypothesis. Resurveys of 72 of Highton's populations in the 2000s found evidence of further demographic declines in several species and sites (Caruso and Lips 2013) as well as body size reduction in several species, supporting potential climate‐change‐driven plasticity (Caruso and Lips 2013). Muletz et al. (2014) investigated if salamander declines at Highton's historic sites in Appalachia (including Indian Grave Gap) coincided with the emergence of the amphibian chytrid pathogen *Batrachochytrium dendrobatidis* (*Bd*) as observed in Central American plethodontids (Cheng et al. 2011). They detected no Bd in *Plethodon* specimens collected during the reported declines (*n* = 892) and suggested that a novel undetected pathogen may instead be driving declines. Thus, the factors underlying these widespread declines remain unclear and the vulnerability of remaining populations to further global change has not yet been assessed.

Here, we use molecular sequence data to test if signals of declines are observable in the genomes of *Plethodon* salamanders. We use an exonic target‐capture approach to generate SNP data for six species of *Plethodon* across three time periods (1960s–1970s, 1980s–1990s, and 2018–2019) that also correspond to different preservation conditions (liver removed from formalin‐fixed morphological vouchers, frozen blood in capillary tubes, and freshly sampled liver tissue preserved in RNAlater, respectively). Our samples are from two sites in central and southern Appalachia, one with documented declines (Indian Grave Gap) and one without (Skull's Gap). We investigate (1) the recoverability of different classes of molecular markers from historical formalin‐fixed and frozen blood‐derived DNA, (2) the impact of bioinformatic regimes on demographic estimates from historical DNA, (3) if *Plethodon* in the two communities show a genetic signal of population decline, and (4) allele frequency changes through time in immune‐related loci.

## 2 Methods

### 2.1 Site Background

**FIGURE 1:**
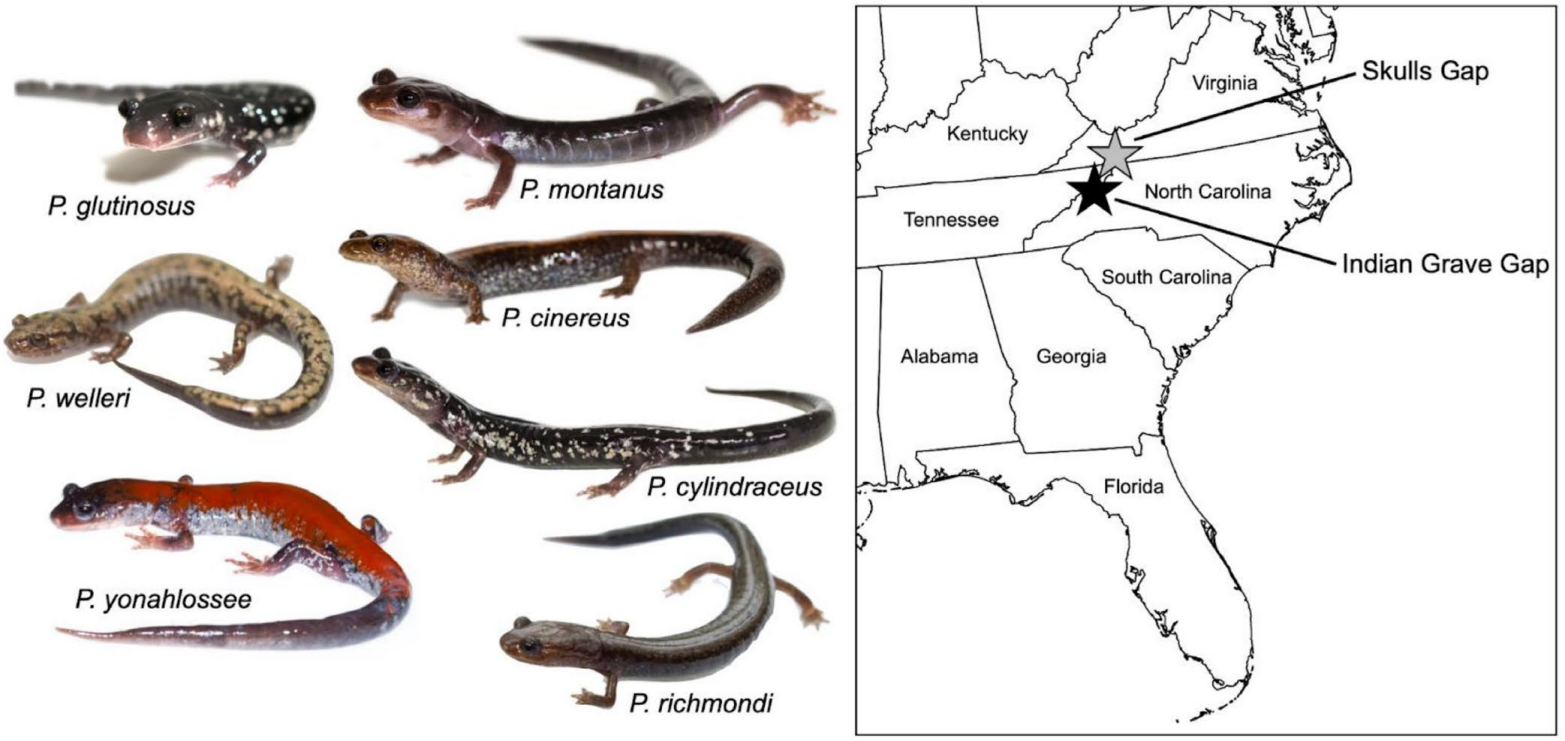
Focal study sites Skull's Gap in Virginia and Indian Grave Gap on the border of North Carolina and Tennessee. Images of focal *Plethodon* species. Map courtesy of Vemaps.com and images courtesy of A. Lopez Torres ( *P. cinereus* , *P. cylindraceus* , *P. montanus* ), B. Gratwicke ( *P. richmondi* , *P. welleri* ), D. Huth ( *P. glutinosus* ), J.P. Lawrence ( *P. yonahlossee* ).

The present study focused on two sites historically surveyed by Richard Highton, chosen for their abundance of samples and high number of total *Plethodon* species present: Skull's Gap and Indian Grave Gap (Figure 1). Skull's Gap is located in central Appalachia, in southern Virginia (36° 41′ 58″ N, 81° 36′ 45″ W), and the community includes five *Plethodon* species ( *P. cinereus* , *P. richmondi* , *P. montanus* , *P. glutinosus* , *P. yonahlossee* ). Skull's Gap was surveyed by Highton 12 times between 1969 and 1993; these survey data showed no evidence of decline in salamanders at this locality. Indian Grave Gap is in southern Appalachia along the border of NC/TN (36° 06′ 36″ N, 82° 21′ 40″ W), and the community includes five *Plethodon* species ( *P. cinereus* , *P. welleri* , *P. cylindraceus, P. montanus*, and *P. yonahlossee* ). Indian Grave Gap showed evidence of population declines in recent surveys (Caruso and Lips 2013) as well as across 17 surveys conducted by Highton from 1970 to 1995.

### 2.2 Sample Selection

We used tissue samples from three time points in our study: liver from formalin‐fixed voucher specimens (historic liver; 1969–1978), historic frozen blood (1984–1993), and modern frozen liver (2018–2019). For both historic and modern samples, depending on the species and/or site, some samples may all have been collected during a single sampling event or across multiple sampling events/years within the corresponding time point (1960s–1970s, 1980s–1990s, 2010s; Tables 1, S2). We harvested liver tissue from 80 formalin‐fixed voucher specimens (largest and most intact specimens from 105 available) from the Amphibian and Reptile collections of the National Museum of Natural History, Smithsonian Institution (USNM), collected between 1969 and 1978 by Richard Highton. As with many specimens from this era, there were no tissue samples from these animals preserved or archived specifically for genetic analysis. We harvested ~30 μg of liver tissue from the morphological vouchers by cutting ~5–10 mm ventrally and extracting liver via forceps. We stored samples in RNA‐later for < 12 h before freezing at −80°C (Table S2). For our second sampling time point (1984–1993), we used 42 blood samples collected by Highton, who stored harvested blood in glass capillary tubes at −70°C after separating the blood cells from plasma for gel electrophoresis. Finally, we collected 89 modern tissue samples for six species ( *P. cinereus* , *P. montanus* , *P. glutinosus* , *P. welleri* , *P. cylindraceus*, and *P. yonahlossee*) at Skull's Gap and Indian Grave Gap on three field excursions in the months of April and June 2018 and September 2019. We followed the same general sampling strategy Richard Highton used in his surveys. This included searching across several acres during the day and flipping cover objects like logs and rocks to collect salamanders sheltering beneath them, as well as surveying along the Appalachian Trail at night and capturing individuals that were active at the surface. Although we do not have unique locality details for each individual collected during Highton's historical surveys, based on our knowledge of his sampling strategy and our own experience at these sites, it is rare to find multiple individuals under the same cover object. While we did collect three *P. richmondi* at Skull's Gap, we collected insufficient numbers of specimens to include them in downstream molecular analyses. Likewise, modern *P. cinereus* samples were dropped during downstream bioinformatic processing (see Methods). Specimens were euthanized using buffered MS‐222 (tricaine methanesulfonate) in doses appropriate for the body size of each individual, recommended as standard practice by the American Society of Ichthyologists and Herpetologists, the Herpetologist's League, and the Society for the Study of Amphibians and Reptiles. Liver tissue was harvested and stored in RNA‐later for ~36 h before being frozen at −80°C. Morphological voucher specimens were preserved in formalin, properly positioned for ease of future data gathering, and deposited at USNM. USNM numbers are included in Table S2.

**TABLE 1:** Summary of samples for each preparation type and those that passed laboratory (QC Lab) and bioinformatic (QC Bio) quality thresholds as described in Section 2.

Time period	Sample type	Skull's Gap			Indian Grave Gap		
		Species	Samples	QC Lab/Bio	Species	Samples	QC Lab/Bio
1969–1978	Historic liver	5	38	38/37	5	49	42/42
1984–1993	Historic frozen blood	2	20	14/12	2	22	21/20
2018–2019	Modern liver	3	40	22/17	4	49	29/21

### 2.3 DNA Extraction

DNA extractions of formalin‐fixed liver tissues followed O'Connell et al. (2022) using a modified salt‐extraction protocol and were performed in a DNA extraction laboratory at the Laboratory of Analytical Biology at the Smithsonian National Museum of Natural History (NMNH). DNA fragment distributions were checked for representative extractions on a 1% agarose gel using 1–5 μL of DNA and all samples were quantified using the QUBIT 2.0 Fluorometer (Life Technologies, Grand Island, NY, USA; results in Table S2). We aimed for fragment sizes > 200 bp. If DNA quantifications were below 2 ng/μL, we conducted multiple extractions of the same sample and pooled the final product. DNA extraction for modern liver tissue and blood capillary samples followed a standard salt‐extraction protocol (Sambrook and Russell 2001) at the NMNH.

### 2.4 Target‐Capture Probe Design

We generated target sequences from several marker types to (1) investigate the effects of population declines on different classes of loci and (2) create a flexible genomic resource for future research on other *Plethodon*‐focused research questions. Our marker sets included genome‐wide exons (from transcripts), phylogenetically‐informative nuclear loci, Ultra Conserved Element (UCE) loci, vision‐related genes, hearing‐related genes, and immune‐related genes. For genome‐wide exonic targets, we used transcript sequences generated from *P. cinereus* (Ellison et al. 2020) to design target loci for capture. We designed probes using custom scripts as follows: We first trimmed the *Ambystoma mexicanum* reference genome (AmexG v3.0.0) to exonic sequence and randomly selected one exon per gene > 200 base pairs (bp) in length. We used blastn v.2.6.0 (Altschul et al. 1990) to identify regions of overlap between our transcripts and the reference exons. We retained BLAST hits between 200 and 400 bp in length with GC content between 30% and 70%. We randomly chose one top BLAST hit per exon and required that selected loci were annotated in the *A. mexicanum* reference genome resulting in a set of 2377 loci. For phylogenetically‐informative loci, we included nine legacy nuclear loci often used in sanger sequencing‐based studies of amphibian phylogenetics (*ALB, BDNF, GAPD, MLC2A, POMC, RAG1, RHO, SLC8A3, V2R*; Portik et al. 2023). For these nine loci, we downloaded sequences from Genbank from *P. cinereus* and included the entire sequence rather than a single exon. When a sequence for *P. cinereus* was not available we used another species of *Plethodon* as reference. For UCEs, we included the 87 most variable loci from a previous study of *Plethodon serratus* (Newman and Austin 2016). To broaden the utility of our probe set, we also included the entire coding sequence from 10 vision‐related genes downloaded from NCBI (*RHO* from *A. mexicanum* , and *LWS, SWS1, SWS2, ARR3, SAG, GNAT1, GNAT2, GRK1, GRK7* from *Nanorana parkerii*) and 10 hearing‐related genes (*KLF9, ALDH1A3, ATOH1, NKX2, OTX2, MSX2, CACNA1A, MHX3, dlx2, tbx2* from *Xenopus laevis*). We included three immune‐related genes by identifying the *MHC* class I alpha chain, class II betachain, and class II exon 2 for *P. cinereus* by mapping our transcripts against amphibian *MHC* sequences (downloaded from NCBI) in Geneious v.9.1.2 (https://www.geneious.com) under default settings. We designed probes based on the consensus sequence of the mapped reads for each of the three *MHC* sequences. We included four other immune‐related sequences that we manually identified from the BLAST output (*TLR‐2*, *CXCR4*, *ILF2*, *ILF3*). Target sequences underwent further filtering and were synthesized by myBaits (Arbor Biosciences, Ann Arbor, MI) using 90 bp probes with 2× tiling. The final probe set resulted in 16,226 baits targeting 2572 markers.

### 2.5 Genomic Library Preparation

Libraries for historic liver (*n* = 80) were prepared by Arbor Biosciences. We prepared genomic libraries of historic blood samples (*n* = 42) and modern liver samples (*n* = 89) at the Center for Conservation Genomics at the Smithsonian's National Zoo & Conservation Biology Institute and NMNH. We first sheared 1–3 μg of DNA per sample to ~350 bp using a Covaris ME220 (Covaris Inc. Woburn, MA, USA), and checked the distribution of fragments for representative samples using an Agilent Tape Station (Agilent Technologies, Santa Clara, CA, USA). Libraries were prepared using SureSelect Library Prep Kits (Agilent Technologies, Santa Clara, CA, USA) with in‐house double‐indexed 8 bp Nextera‐style adapters. Libraries were amplified in two 50‐μL reactions using Herculase II Fusion DNA Polymerase using 13–16 cycles (Table S2) and prepared by hand or with an Apollo 324 robot in batches of 24 samples. We sent these libraries to Arbor Biosciences for quality control and hybridization reactions. In qPCR‐based checks conducted by Arbor Biosciences, several of the historic blood and modern liver libraries failed qPCR checks, indicating that barcode indexing had failed in these samples (despite the presence of adequate genomic DNA); thus, we moved forward with all 80 historic liver, 32/42 frozen blood, and 43/80 modern liver genomic libraries for capture reactions (Table S2). Hybridization reactions followed the protocol of the myBaits v4 manual. Paired end 150 bp sequencing was conducted by Arbor Biosciences on 8% of an Illumina Novaseq S4 lane.

### 2.6 Genomic Data Processing

We processed sequence data using the Python pipeline SECAPR v.1.1.15 (Andermann et al. 2018), an extension of the Phyluce pipeline (Faircloth 2016), as well as our custom Python scripts (Data Availability). We first screened raw reads for contamination using FastQ Screen v.0.14.1 (Wingett and Andrews 2018) by calculating the proportion of raw reads that mapped to either the human genome (GRCh38) or a database of representative bacterial genomes (11 k + genomes) from the NCBI RefSeq database using bowtie2 v.2.3.5 (Langmead and Salzberg 2012). We used FastQ Screen (‐no hits command) to remove reads that mapped to either the human or bacterial genomes and cleaned filtered reads using Trim Galore! v.0.6.4 (retain unpaired; Krueger 2012). We used bwa mem to map cleaned reads to a mitochondrial genome reference of *P. montanus* , but did not recover suitable fragments to move forward with mtDNA analyses (indicating low levels of capture bycatch).

We conducted a preliminary run of our whole bioinformatic pipeline using species‐specific references and found that *P. yonahlossee* samples generated the most complete contig assemblies and subsequent read mapping. Thus, we created a pseudo‐reference from eight *P. yonahlossee* frozen liver samples by assembling contigs with Abyss v.1.3.7 (Simpson et al. 2009) (kmer = 90) and used this reference for downstream analyses. We matched assembled contigs to target locus sequences using the “secapr find_target_contigs” command with min coverage = 60, min‐identity = 80 and the “keep‐paralogs” flag. We aligned recovered markers using MAFFT v.7.130b (Katoh and Standley 2013) (no‐trim and ambiguous flags; Katoh et al. 2002) and cleaned alignments following (O'Connell et al. 2022), then created a consensus of each alignment using the “cons” command (plurality 0.1 and setcase 0.1) from EMBOSS run within the secapr reference_assembly script. Using custom Python scripts, we filled in loci missing from our new consensus reference with sequence from the original target loci. Based on the output of the “find_target_contigs” step, we removed putative paralogs from our reference, requiring at least three contigs to map to a locus in at least 50% of the samples. We mapped the cleaned reads of all samples to this new reference (mean length 710 bp) using the “reference_assembly” script (min coverage = 6×). Preliminary phylogenetic analysis suggested that seven samples were contaminated or misidentified with another *Plethodon* species, including five modern liver samples and two historic liver samples. Thus, we continued subsequent analyses with 149 samples, including 79 historic liver, 32 frozen blood, and 38 modern liver samples.

We corrected for formalin‐induced deamination using mapDamage2 v.2.0.6 (Jónsson et al. 2013), a computational framework that tracks and quantifies DNA‐damage patterns in ancient and historical DNA sequences. To quantify nucleotide damage, we calculated the average rate of C > T and G > A misincorporations for each data type across the first 25 bp of each read. We called SNPs using BCFtools v.1.9 (Danecek et al. 2011), using the mpileup command with maximum coverage of 1000×, calling SNPs from both reads (‐A). We followed (O'Connell et al. 2022) to prune putative SNPs within 3 bp of an indel and remove clusters of indels with five or fewer bp between them. For the whole dataset (all species included), we used VCFtools v.0.1.16 (34) to remove SNPs with quality scores < 30, non‐biallelic SNPs, minor allele frequency ≤ 0.015, minor allele count < 3, and minimum coverage < 5×. We further filtered for allele balance (AB > 0.25 and AB < 0.75) and required at least 50% of individuals present at a site, producing a dataset of 31,049 high‐quality SNPs across all samples and full‐length loci. Finally, we chose one random SNP from each retained locus (2229/2572), resulting in a matrix of 141 samples with 2229 unlinked SNPs and 15.7% missing data (calculated following (De Medeiros and Farrell 2018)). We refer to these data as the “all reads” dataset (Table S3).

Preliminary data exploration revealed potential biases in estimates of genetic diversity based on sample coverage. To explore the effect of coverage on diversity estimation (particularly for historical samples), we used Samtools (samtools view ‐bs) to downsample each sample to a mean depth of 24×, then reran the SNP calling pipeline described above, resulting in a dataset we refer to as “downsampled.” Finally, we identified coding/non‐coding boundaries by mapping our original exonic targets to our expanded target fastas (see above) using bwa mem, converted the subsequent outputs to a file in BED format, then used VCFtools to partition our “all reads” and “downsampled” datasets to “coding” and “non‐coding” datasets. Although we targeted exonic regions, bycatch of adjacent sequences is common in target‐capture studies (Faircloth et al. 2012; Portik et al. 2016). Because of monomorphic non‐coding sequences, these datasets yielded fewer SNPs than the all sites (coding + non‐coding sequence) data. Our final filtering resulted in six datasets: (1) all reads and all sites, (2) all reads and non‐coding sites, (3) all reads and coding sites, (4) downsampled and all sites, (5) downsampled and non‐coding sites, and (6) downsampled and coding sites.

We conducted several quality control analyses. First, to ensure that samples clustered by species rather than by sample type, we conducted a principal component analysis in R using adegenet v2.1.10 (Jombart 2008). Second, we examined the effect of sample size on Pi estimates using a regression. Finally, we evaluated if the timespan over which samples were collected within one of the given time periods impacted estimates of diversity. For example, one species/time period/locality combination may have 10 samples all collected in 1985, while another could have 10 samples collected across 1985, 1986, and 1990. To evaluate whether this variation in sampling impacted our estimates of genetic diversity, we regressed median estimates of Pi for all permutations for a given population against the total number of years sampled as well as the range between the first and last year sampled (timespan). In the example above (1985, 1986, and 1990), total number of years sampled would be three and the timespan would be five.

### 2.7 Estimating Genetic Diversity and Historical Demography Across Genetic Marker Types

Across all datasets, we investigated changes in genetic diversity by estimating several population genetic statistics between populations and time points, including nucleotide diversity, Watterson's theta, and Tajima's D. Nucleotide diversity and Watterson's theta measure genetic variation within a population, with declining values through time supporting bottlenecks or population declines and increasing values supporting stable or expanding population sizes. Tajima's D compares these two statistics to identify demographic shifts, with negative values supporting expansion and positive values indicating bottlenecks or balancing selection. Declining Tajima's D through time therefore supports stable or expanding populations, while increasing Tajima's D through time suggests bottlenecks or balancing selection.

We accounted for uneven sample sizes across time points by implementing permutation tests (O'Connell et al. 2018). We randomly subsampled the data for each replicate type to a minimum (three) and maximum (all available samples) number of samples using vcftools v0.1.16 and estimated nucleotide diversity, estimated and observed heterozygosity, and private alleles using the populations module in STACKS v.2.54 (Catchen et al. 2013). For each species and site, we tested for significant differences in nucleotide diversity (pi) between time points using an ANOVA where we compared nucleotide diversity for each species at each site and time period. In cases of significant interactions, we conducted post hoc tests to explore pairwise differences using the TukeyHSD function in R. To calculate Watterson's θ and Tajima's D, we used scikit‐allel v.1.3.11 (Miles et al. 2024) to parse the single‐SNP VCF and calculated allele counts and segregating sites in 150 bp sliding windows. Because mutation rate (per site, per generation) is unknown in amphibians, we used the mean rate for fishes of 5.97e‐09 (Bergeron et al. 2023). We repeated this procedure 20 times and plotted the distribution using R (R Core Team 2021). We calculated significant values of Tajima's *D* by testing if the mean estimate across permutations was significantly different from neutrality (*D* = 0) using critical values of |*D*| > 1.5 as evidence for selection, corresponding approximately to *p* < 0.05 under neutral expectations. We tested for significant values of Watterson's Theta by calculating the coefficient of variation across replicates with CV > 1.0 indicating significant variability in diversity patterns. We also estimated nucleotide diversity for the three immune loci that passed bioinformatic filtering (*CXCR4, ILF2, ILF3*; 49 variable sites from three loci), for phylogenetic loci (308 variable sites from nine loci), and for UCEs (33 variable sites from nine loci). We also calculated allele frequencies for all immune‐related SNPs. To differentiate these allele shifts from background demographic processes (e.g., drift or bottleneck), we applied a genome‐wide Fst outlier test (Lewontin and Krakauer 1973). First, we calculated a neutral genome‐wide Weir–Cockerham Fst rate from our non‐coding SNP data (the 2811 SNPs in the flanking sequence of the exon loci), and then we calculated Fst for the 29 immune‐related SNPs and identified sites with Fst greater than two standard deviations above the mean.

## 3 Results

### 3.1 Capture Statistics by Locus Type

Our target loci included a variety of locus types (e.g., UCEs, commonly used phylogenetic markers, immune genes) in addition to the random exons selected from our reference transcriptome (Table 2). In the “all reads” dataset, we recovered 9/9 nuclear “phylogenetic” loci with a mean depth of 141× coverage, but had much lower success with the other locus types: 9/87 UCE loci with 21.5× coverage, 3/7 immune loci with 120× coverage, 2/10 vision‐related loci with 60× coverage, and 3/10 hearing‐related loci with 20.4× coverage. We retained 2229 of 2572 of the targeted random exons with 70.9× mean coverage.

**TABLE 2:** Summary statistics of capture success following bioinformatic processing for each marker type.

Locus set	# Loci	Mean length	“all reads”		“downsampled”	
			Passed filtering	Mean depth	Passed filtering	Mean depth
Random exons	2572	747	2050	70.9	1577	80.0
Phylogenetic	9	895	9	141.1	8	149.0
UCEs	87	570	9	21.5	3	40.8
Vision genes	10	550	2	60.0	1	42.2
Hearing genes	10	614	3	20.4	2	23.7
Immune genes	7	840	3	119.8	3	33.6

### 3.2 Dataset Completeness by Filtering Regime

The “all reads all sites” dataset had the greatest number of loci (2229 of 2572 original targets), with an average of 57× read depth, 13.9 sites per locus, and 86.7% variable loci (Table S3). When we filtered to only coding sites, we recovered almost all of the loci (2222), read depth increased to 61.1×, with 12.1 sites per locus, and 86.4% variable sites, generally very similar to the full dataset. However, when we filtered to non‐coding sites, we only retained 1295 loci with a mean depth of 31.3×, 3.2 sites per locus, and only 50.0% variable loci. We initially expected flanking (non‐coding) regions to exhibit greater variation than coding sequence but found that lower read depth and higher levels of missing data led to most of the variation in these sequences being filtered out during bioinformatic processing. Deeper sequencing would likely improve assembly and retention of variation in flanking regions.

In the downsampled datasets, our complete locus dataset (all sites) retained 2091 loci with mean depth of 32.2×, 12.0 sites per locus, and 81.3% variable sites. The downsampled coding dataset retained 2083 loci with a mean depth of 33.4×, 10.9 sites per locus, and 81.0% variable sites. Similar to the “all reads” data, the non‐coding downsampled dataset exhibited much less variation, with 888 loci retained with mean depth of 20×, 2.6 sites per locus, and 34.0% variable sites, suggesting again that lower coverage of flanking sequences filtered out a majority of variation for these regions.

### 3.3 Capture Statistics by Replicate Type

General statistics for all samples by sample type are given in Table 1, Table S2 and summarized here. Mean number of cleaned reads exhibited minor variation between sample types, with an average of 6.7 million reads for historic liver, 5.7 million reads for frozen blood, and 7.5 million reads for modern liver samples. Exogenous contamination was higher in historic liver, with a mean level of contamination of 18.4% (SD = 16.72%). Mean coverage exhibited wide standard deviation before downsampling and was highest for modern liver samples (54×, SD = 33.6×), followed by historic blood (37.7×, SD = 25.8×) and historic liver (19.2×, SD = 16.7×). Following downsampling, mean coverage in frozen liver samples dropped to 21.9× (SD = 12.2), blood to 21.8× (SD = 8.8), and increased on average in historic liver to 26.2× (SD = 16.3×) because subsequent filtering dropped several lower‐coverage samples. Nucleotide damage estimates for formalin‐fixed historic liver samples estimated by mapDamage2 were generally low 3′ G > A = 0.006 (SD = 0.005), and 5′ C > T = 0.008 (SD = 0.006). Sample types yielded similar proportions of total SNPs in the final “all sites” dataset, with historic liver retaining 79.8% of total SNPs, historic blood 71.0%, and modern liver 88.0%. Our PCA indicated that samples clustered by species/population rather than by replicate type (Figures S1 and S2).

### 3.4 Estimates of Genetic Diversity by Dataset

**FIGURE 2:**
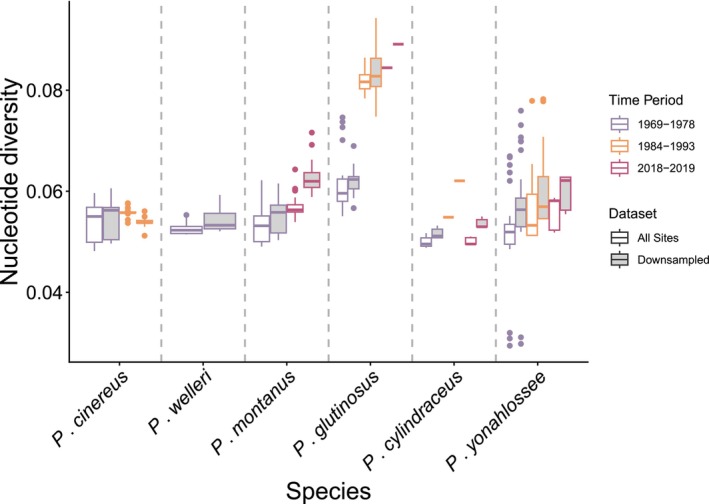
Estimates of genetic diversity for each species comparing the “all reads all sites” to the “downsampled all sites” datasets. Colors correspond to time period and tissue preparation type, with purple as formalin‐fixed historic liver samples (1960s–1970s), orange as historic frozen blood samples (1980s–1990s), and maroon as modern liver (2018–2019). Datasets are shown by fill color, with “all reads all sites” in white and “downsampled all sites” in gray. The plot shows that diversity estimates are slightly higher for most species and tissue preparation types in the “downsampled all sites” dataset, and that the direction of changes are largely consistent.

We compared estimates of nucleotide diversity (pi) across our six datasets: all reads all sites, all reads coding sites, all reads non‐coding sites, downsampled all sites, downsampled coding sites, and downsampled non‐coding sites. First, we evaluated the effect of downsampling on estimates of diversity and found that estimates of pi were higher with downsampled data and the direction of change was consistent for most species (Figure 2). The one exception was *P. cinereus*, where downsampled estimates of pi for frozen blood samples were lower than the “all reads” dataset. We also compared estimates of pi using all SNPs compared with single SNPs for the “all reads” and downsampled datasets and found again that the direction of change was consistent across datasets but that estimates were lower for the “single‐SNP” data compared with using all SNPs (Figure S3). Finally, we compared coding to non‐coding (flanking)‐derived SNPs and found a similar trend in that the direction of change was consistent for most species, but that absolute estimates of diversity were higher for coding sites (Figures S4 and S5). Given the consistency in the direction of relative change in pi through time, we focused all subsequent analyses on the “all reads all snps” dataset.

**FIGURE 3:**
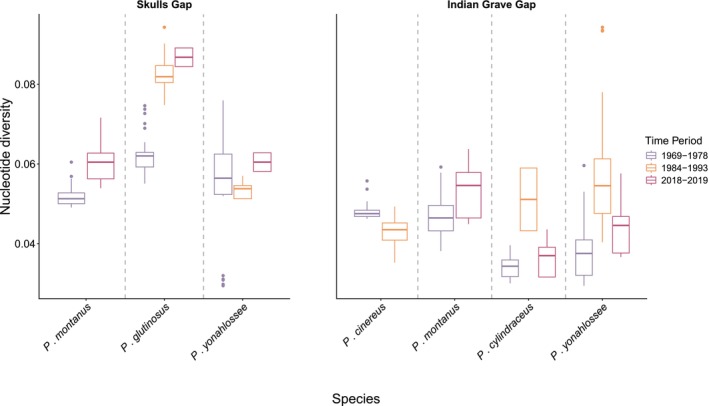
Estimates of genetic diversity for each species (vertical bottom labels, excluding *P. welleri* due to sampling) and locality based on the “all reads all sites” dataset. Colors correspond to time period and tissue preparation type, with purple as formalin‐fixed historic liver samples (1960s–1970s), orange as historic frozen blood samples (1980s–1990s), and maroon as modern liver samples (2018–2019). At Skull's Gap (no reported declines), nucleotide diversity increased over time for all three species with temporal sampling. At Indian Gap Grave (reported declines), changes through time were more idiosyncratic for the four species with temporal sampling.

When we investigated the effects of sample size on estimates of nucleotide diversity using permutation tests, we found that the largest variation of estimates was for populations with small sample sizes and that values of pi converged across estimates with larger numbers of samples (Figure S6). For most species and replicates, estimates of pi were robust (small standard deviation based on bootstrap technique described in Methods) to changes in sample size, but some, such as *P. yonahlossee* estimates for historic liver (*n* = 10) exhibited a wide standard deviation (Figure 3). Finally, when we evaluated the effect of sampling years on genetic diversity, we found that the distribution of Pi across populations was similar for all multi‐year collections, but highest in *P. glutinosus* , which was sampled in a single year for both historic liver and historic frozen blood (Figure S7).

### 3.5 Site‐ and Species‐Specific Demographic History

In the face of recent population bottlenecks, we would expect to find reduced nucleotide diversity and Watterson's Theta through time (fewer polymorphisms) and modestly positive Tajima' SD > 0 driven by an excess of intermediate‐frequency alleles across marker types. At Skull's Gap, for which prior field surveys did not find evidence of demographic declines, we found evidence of expansion in all three species, supported by increasing Pi and Watterson's Theta and declining Tajima's D. Thus, overall our data are consistent with reports of stable demography at Skull's Gap. By contrast, at Indian Grave Gap we observed idiosyncratic patterns across species. First, we observed evidence of decreased nucleotide diversity, increased Watterson's theta, and increased Tajima's D for *P. cinereus* between the 1960s–1970s and 1980s–1990s (unfortunately samples for the most recent time period did not pass lab QC), consistent with sudden population contraction. Meanwhile, the other three species showed mixed evidence for expansion and bottlenecks, suggesting that at these recent time scales it can be very difficult to differentiate between demographic scenarios, particularly with small sample sizes.

**FIGURE 4:**
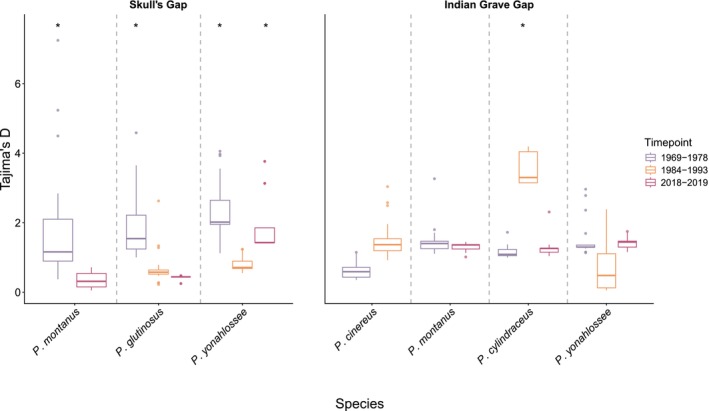
Estimates of Tajima's D for all species at Skull's Gap (SG) and Indian Grave Gap (IGG). Colors correspond to time period, with purple as historic liver samples (1960s–1970s), orange as frozen blood (1980s–1990s), and maroon as frozen liver (2018–2019). Timepoints significantly different from neutrality are shown with asterisks above box plots. Increasing values through time support population declines or balancing selection, while decreasing values support stable or expanding populations.

At Skull's Gap, we found evidence of expansion in all three species, as indicated by increasing nucleotide diversity over time for *P. montanus* and *P. glutinosus* for the full dataset, as well as for phylogenetic and immune loci (Figure 3; Figure S8). With UCEs, *P. montanus* also exhibited increasing diversity, while for *P. glutinosus* diversity at UCE loci decreased slightly (Figure S8). For both species, estimates of Watterson's Theta increased, while Tajima's D decreased but remained greater than zero, suggesting ongoing expansion (Figure 4, Figure S9). For *P. yonahlossee*, we found more varied patterns, including an initial decrease followed by a slight increase in Tajima's *D* (Figure 4). Differences in nucleotide diversity estimated from the “all reads all sites” dataset were significantly different in *P. glutinosus* between the 1960s–1970s and 2018–2019 (adj. *p* = 0.0001) and 1980s–1990s and 2018–2019 (adj. *p* = 0.00), and in *P. yonahlossee* between 1960s–1970s and 1980s–1990s (adj. *p* = 0.00), and 1980s–1990s and 2018–2019 (adj. *p* = 0.00) (Table S4). No Watterson's Theta values were significantly different from a neutral expectation, whereas Tajima's D values were significantly different from neutral (indicating balancing selection) in *P. glutinosus* , *P. montanus* , and *P. yonahlossee* during the 1960s–1970s and in *P. yonahlossee* during 2018–2019 (Figure 4, Figure S9).

At Indian Grave Gap, patterns were more idiosyncratic across species. For *P. cinereus* , we found decreasing nucleotide diversity in the “all sites” and phylogenetic datasets but increases in the UCEs and immune loci (Figure 3, Figure S8), as well as decreases in Watterson's Theta and Tajima's D between the 1960s–1970s and 1980s–1990s, generally suggesting declines or a bottleneck (Figure 4; Figure S9). With *P. montanus* we found increasing nucleotide diversity for “all sites,” phylogenetic, and immune loci, and Watterson's Theta, but decreases in UCEs and Tajima's D, supporting stable or expanding population sizes. In *P. cylindraceus* we observed increasing nucleotide diversity for “all sites,” phylogenetic loci, and immune loci (with a peak in 1980s–1990s) as well as Watterson's Theta, and declines in UCEs, and Tajima's D, again with a peak in the 1980s–1990s and decline thereafter (Figures 3 and 4; Figures S8 and S9), supporting a potential decline in this species from the 1960s–1980s but expansion thereafter. Finally, for *P. yonahlossee* we found increasing nucleotide diversity for the “all sites” data, phylogenetic loci, UCEs, immune loci and Watterson's Theta (with a peak in 1980s–1990s for “all sites,” immune loci and Watterson's Theta), and a decline in Tajima's D, with a decrease in the 1980s–1990s for UCEs and Tajima's D (Figures 3 and 4; Figure S9). No Watterson's Theta values were significantly different from a neutral expectation, whereas Tajimas D values were significantly different from neutral (suggesting balancing selection) in *P. cylindraceus* in the 1980s–1990s (Figure 4; Figure S9).

With immune loci, we found much lower nucleotide diversity in *P. cinereus* and *P. montanus* compared with the other two species, with *P. cinereus* exhibiting allele fixation in all three loci in the 1969–1978 samples (Figure S8). Furthermore, in *P. cylindraceus* and *P. yonahlossee* , coding sites indicated an increase in diversity through time, whereas non‐coding sites supported declines through time (Figure S4). We manually inspected each of these aberrant datasets and did not observe significant deviations from the mean in metrics such as sample sizes, coverage, or number of SNPs. Our sampling for Indian Grave Gap included all three time points for two species, *P. cylindraceus* and *P. yonahlossee* . In both cases, estimates of nucleotide diversity were significantly different between all three time points (Table S4).

### 3.6 Allele Frequency Changes in Immune Loci

**FIGURE 5:**
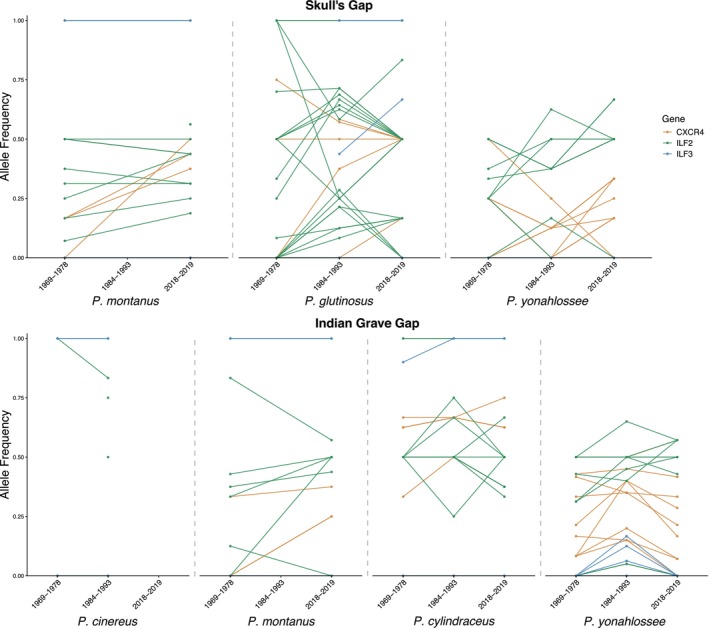
Allele frequencies through time for immune‐related loci. Colors correspond to locus; not all three time periods were sampled for each species. In cases where only a single dot is present, one or more timepoints lack data for that allele due to poor capture or bioinformatics QC.

We identified 49 unique innate immune‐related variants across all individuals, with 13 SNPs in CXCR4, 30 in ILF2, and six in ILF3 (Table 3). No sites were private to any species and site, with all SNPs shared by most species. Baseline mean Fst for SNPs in exon flanking sequence (non‐coding) was 0.345 while mean immune‐related Fst was 0.462 (34% higher than neutral baseline). Nineteen SNPs exhibited large changes in allele frequency over time (11 from ILF3 and 8 from CXCR4); however, only one of these from ILF3 was also identified as an Fst outlier SNP. This SNP was shared by *P. cinereus* at both sites and *P. yonahlossee* from Indian Grave Gap. Percent heterozygosity across these polymorphic sites was relatively high for all species except *P. cinereus* , which was only 5.3% heterozygous compared with a mean of 26% for all other species. We also identified several alleles that were fixed or at very high frequencies (Figure 5). In particular, of the 37 SNPs present for *P. cinereus* , 84% were fixed and 19% had unique alleles not present in other species. *Plethodon cinereus* was also missing 31 alleles present in other species, indicating either incomplete capture or a loss of allelic diversity.

**TABLE 3:** Summary of allele variation in immune‐related loci. Summaries for each species reflect combined samples across all time points and populations.

Species	Sample size	Total SNPs	Polymorphic SNPs	% Polymorphic	CXCR4	ILF2	ILF3
*P. yonahlossee*	40	49	24	49.0%	9	12	3
*P. glutinosus*	20	49	22	44.9%	5	16	1
*P. montanus*	34	49	14	28.6%	4	10	0
*P. cylindraceus*	12	49	13	26.5%	4	8	1
*P. cinereus*	26	37	6	16.2%	2	3	1
*P. welleri*	9	32	5	15.6%	3	2	0

## 4 Discussion

### 4.1 Reliability of Historical Samples for Estimates of Genetic Diversity

The use of historic DNA, including that derived from liver extracted from morphological voucher specimens, is a rapidly growing field becoming distinct from the field of ancient DNA (Raxworthy and Smith 2021). Despite recent advances in DNA extraction (see Table 1 in Raxworthy and Smith 2021) and bioinformatic pipelines (Oliva et al. 2021), the effects of specimen fixation upon DNA quality and how this impacts the completeness and accuracy of historic DNA datasets is still not fully understood. Particularly for historic liver, and especially for older specimens, the length of time spent in formalin and other conservation conditions that impact DNA quantity and integrity were often not recorded by the original collectors or as part of standard collections management practice. For instance, factors such as exposure to heat or light may further contribute to DNA degradation. As we better understand the effects of formalin (and other environmental conditions specific to museum specimens) on DNA degradation, the community needs better models for correcting DNA‐damage‐induced biases in historical DNA as compared with ancient or paraffin‐embedded DNA (Haile et al. 2019; Raxworthy & Smith, 39). For example, in this study, and in (O'Connell et al. 2022) we used mapDamage2, which reported very low levels of DNA damage (3′ G > A = 0.006 [SD = 0.005], and 5′ C > T = 0.008 [SD = 0.006]); this may indicate that models developed for ancient DNA may not be well suited to modeling degradation patterns of historic liver samples. Conversely, these results may indicate that DNA degradation in historic liver samples is low and unlikely to bias downstream analyses.

In the present study, we can make several observations about the utility of historic tissue for population genetic inference. In the absence of demographic change, we would expect a lower baseline estimate of diversity for historic samples because of a documented homozygous bias (Ewart et al. 2019) and as such, patterns of increasing diversity such as we observed in species at both sites may be more indicative of stable diversity through time rather than true increases in diversity. Correspondingly, a homozygous bias in historic samples lends greater support to any observed decreases in diversity between time points, such as those we observed in *P. cinereus* at Indian Grave Gap. Interestingly, statistics estimated from blood samples collected in the 1980s–1990s gave wider estimates in variation between individuals compared with the other two time points (e.g., Figure 3). Other blood samples from the Highton collection were used for genetic studies of Spring Salamanders (*Gyrinophilus* spp.) using ddRADseq (Grant et al. 2022) along with ethanol‐preserved modern tissues with no batch effects observed, suggesting that DNA quality among these sample types (historic and fresh liver vs. blood) should be similar. Estimates of genetic diversity can be impacted by a variety of artifacts related to both wet lab (e.g., PCR cycles or library prep batch effects (Yu et al. 2024)) and bioinformatic processing (e.g., missing data thresholds), but our blood samples were processed alongside the fresh samples in the wet lab and we applied the same criteria to all samples in our bioinformatic pipelines. The high variation in blood samples suggests the potential for a base composition artifact in the frozen blood samples, which could be explained by their storage in heparinized tubes. Heparin is known to lead to PCR inhibition, which could introduce biases in downstream analyses (Pyron et al. 2024). However, PCR is only used at the indexing step for our library preparation and would have only introduced bias at indexing. Future research using replicate testing from the same individuals would be helpful to better understand if blood preserved in heparinized tubes produces biased genetic diversity estimates relative to frozen tissue samples.

Finally, we evaluated the impact of sampling timespan on estimates of demography. Depending on the availability of historical samples, it may not always be possible to obtain a sufficient sample size of individuals to represent a historical time point without spanning across multiple sampling events or years. In the present study, our samples within a given time point were collected over a period of 1 year up to 11 years. Sampling across multiple years may span several generations depending on the species, which could influence estimates of genetic diversity and other population genetic parameters. However, when we plotted the median value across permutation estimates for each population, we obtained similar distributions for populations sampled in a single year or across multiple years, suggesting this had a negligible impact in the present study (Figure S7).

### 4.2 Impacts of Bioinformatic Decisions on Demographic Estimates

In addition to capturing exons and commonly used phylogenetic markers for population inference, we tested the efficacy of capturing other types of loci related to hearing, vision, UCEs, and immune response. While our bioinformatics pipeline performed well for capturing exonic targets, other targeted loci performed poorly. This discrepancy may be due to a few factors. First, capture efficacy may have been poor due to phylogenetic distance with the target sequences: *Plethodon* sequences were not available for these other types of loci and thus for some loci we designed targets from *Ambystoma* salamanders (~130 mya divergent) and *Xenopus* (~275 mya divergent from *Plethodon*). Increased tiling of probes during capture design could reduce these biases, but as more amphibian genomes become publicly available (Pyron et al. 2024; Womack et al. 2022) phylogenetic distance will become less of a concern even for under‐represented taxonomic groups. Alternatively, these loci may have hybridized well during the wet‐lab experiment, but not passed bioinformatic filtering. A bioinformatic pipeline that directly maps loci of interest may have been more successful (Schott et al. 2017). As such, for studies focused on questions of phylogeny or demography, we recommend targeting exons (as opposed to UCEs for example) generated from a reference genome or transcriptome of a closely related species because these performed well and exhibited high rates of variation per locus in our data set. However, given many population genetic statistics assume loci are evolving neutrally, future studies could also consider randomly sampling the genome using probes designed from a RADseq dataset, an approach which has also been demonstrated to effectively capture loci from historical specimens (Jensen et al. 2018; Suchan et al. 2022; O'Connell et al. 2022). While the nine phylogenetic loci also captured well and could be included for phylogenetic‐focused studies, using these loci in isolation provided less power for the population‐specific questions we were investigating in this study. Finally, studies focused on specific groups of loci like the hearing or vision genes we included would benefit from focusing sequencing efforts to boost total read count on those loci as opposed to including them with a cohort of other loci. Several of these loci “dropped out” during bioinformatic QC due to low coverage.

In the first stages of our bioinformatic pipeline, we identified significant differences in coverage between sample types, with modern samples having much higher coverage than historic liver, possibly due to higher rates of exogenous contamination in the historical samples. We hypothesized that differences in coverage would impact estimates of diversity; however, we found that diversity estimates from downsampled data were higher on average (Figure 2). Although downsampling did address potential biases introduced by coverage disparities, several lower‐coverage samples and many sites were filtered out, in turn introducing additional biases due to smaller sample size and reduced coverage. While absolute estimates of diversity were different between datasets, the directionality of changes (increasing or decreasing diversity) was consistent. Thus, we recommend not downsampling if sample sizes will be affected negatively.

Finally, we compared genomic variation between coding and non‐coding sites for all reads and downsampled datasets. In both cases, we recovered substantially more variation in the coding compared with non‐coding sites. This was surprising given the differences in selection between coding and non‐coding sites in the genome and that many studies seek to use variation in the “flanking sequence” of exons for phylogenetic and population genetic inference (Faircloth et al. 2012; Portik et al. 2016). We present a few possible explanations for this pattern. First, we marked exon/intron boundaries using the anuran reference from *Xenopus*, but the large phylogenetic distance between this reference and our focal species may have led to inaccurate classifications of our SNPs as coding or non‐coding. Second, because non‐coding sites were not targeted directly by probes, their coverage was much lower overall, leading to less variation and more missing data relative to coding sites. Although patterns of reduced genetic diversity were more pronounced in non‐coding sites, the reliability of these data for accurately inferring demographic trends is questionable given high levels of missing data and variability of coverage. As more amphibian genomes become available (Pyron et al. 2024; Womack et al. 2022), target loci can more easily be developed from non‐coding genomic regions which would enable more robust comparisons of demographic signals derived from different locus types.

### 4.3 Demographic Changes in Woodland Salamanders

Historical survey data by Richard Highton provided evidence that *Plethodon* populations declined across many sites in the Appalachian Mountains (Highton 2005). Later studies found declines in certain species (though not entire communities) using widespread survey data (Caruso and Lips 2013), as well as evidence of body size reduction through time, consistent with a plastic response to climate change (Caruso et al. 2014). These studies in turn generated critique regarding both the methods that were employed and conclusions drawn from the results (Grant 2015); namely that detection probability (determined by surface activity) of individuals and age classes of *Plethodon* is strongly impacted by local conditions when surveys are conducted and that this had not been accounted for (Connette et al. 2015; Gade and Peterman 2019; Gade et al. 2020; Heatwole 1962; Peterman and Semlitsch 2013; Wyman and Hawksley‐Lescault 1987). Further, Grant (2015) argued that the non‐random nature of Highton's historical sampling did not provide an accurate baseline for comparisons of abundance through time. These concerns illustrate the complexity of inferring population changes from non‐standardized survey occurrence data or phenotypic measurements of museum specimens (Link and Nichols 1994; Skelly et al. 2003; Schmidt et al. 2013; Thompson III and La Sorte 2008). Our molecular approach to investigating demographic change in this system provides a new perspective on how population sizes may have changed as well as the potential genomic consequences of those changes.

In the face of recent population bottlenecks, we would expect to find reduced nucleotide diversity and Watterson's Theta through time (fewer polymorphisms) and modestly positive Tajima'SD> 0 driven by an excess of intermediate‐frequency alleles across marker types. At Skull's Gap, for which prior field surveys did not find evidence of demographic declines, we found evidence of expansion in all three species, supported by increasing Pi and Watterson's Theta and declining Tajima's D. Thus, overall our data are consistent with reports of stable demography at Skull's Gap. By contrast, at Indian Grave Gap we observed idiosyncratic patterns across species. First, we observed evidence of decreased nucleotide diversity, increased Watterson's theta, and increased Tajima's D for *P. cinereus* between the 1960s–1970s and 1980s–1990s (unfortunately samples for the most recent time period did not pass lab QC), consistent with sudden population contraction. Meanwhile, the other three species showed mixed evidence for expansion and bottlenecks, suggesting that at these recent time scales it can be very difficult to differentiate between demographic scenarios, particularly with small sample sizes.

Even without strong evidence of synchronous, dramatic declines in genome‐wide genetic diversity across all species at Indian Grave Gap, we found some evidence that salamanders may have been adapting to a changing environment over the last ~50 years. In particular, we found that immune‐related loci exhibited elevated Fst relative to the background genome for all species, which could reflect balancing selection driven by environmental pressures (Hedrick 2002). The patterns of heterozygosity we identified are consistent with trans‐species polymorphism, where balancing selection maintains shared alleles across related species to confer an advantage against potential pathogenic threats (Azevedo et al. 2015; Těšický and Vinkler 2015). This mechanism would also explain why we only identified one Fst outlier in the immune‐related loci; all loci are experiencing a high level of baseline selection. For most of the *Plethodon* we sampled, we found no signal of species‐specific or site‐specific alleles. The one exception was *P. cinereus* , for which a majority of immune‐related alleles were both fixed and unique to the species. Contrary to the pattern of balancing selection observed in the other species, these fixed alleles are more suggestive of a historic bottleneck (also supported by nucleotide diversity, Figure 3) that erased ancestral polymorphism, potentially limiting the ability of *P. cinereus* at Indian Gap Grave (and perhaps elsewhere) to adapt to future pathogen pressures (Sommer 2005). These findings differ from Caruso and Lips (2013), who found that the *P. glutinosus* phylogenetic complex declined the most, but only observed moderate declines at some sampled sites for species in the *P. cinereus* phylogenetic complex.

Losses in genetic diversity scale with the duration and magnitude of a population bottleneck (Nei et al. 1975). For instance, serial sampling revealed substantial loss in genomic diversity in the critically endangered orange‐bellied parrot ( *Neophema chrysogaster* ) over the last ~200 years (Bulgin et al. 2003), but this species has experienced multiple, severe population crashes. As *Plethodon* are so abundant to begin with, dramatic and sustained declines in effective (and census) population sizes would likely be necessary to observe substantive decreases in genetic diversity over a short, decadal time period (Bi et al. 2013). Even with ideal sampling and broader geographic representation, however, it remains an open question if genomic data can capture a signal of declines at such recent time scales, particularly because measurable differences in genetic diversity may “lag” behind reductions in population size due to retention of ancestral alleles (Bi et al. 2013; Bulgin et al. 2003; Jensen et al. 2018). Likewise, recent signals of change in genetic diversity can be affected by elements of life history such as generation times and mutation rates (Van Der Valk et al. 2019) and be overshadowed by historical demography (Dierickx et al. 2020). Although we did not find evidence that proposed declines in woodland salamanders negatively impacted standing genetic diversity within populations (with the possible exception of *P. cinereus and P. cylindraceus*), the sheer abundance of *Plethodon* in Appalachian ecosystems (Caruso and Lips 2013; Caruso et al. 2014) gives them an outsized role in the trophic ecology of those systems, and thus even minor changes in their abundance could have cascading impacts throughout the ecosystem.

Although this study represents an important proof of concept for utilizing genomic data from formalin‐fixed specimens for population‐level research questions, future research could scale up our approach by sampling more individuals per population (20–30 per species) and including additional serially sampled sites. This would improve the accuracy of diversity estimates and clarify the extent to which populations are experiencing shifts in genetic diversity across their respective ranges.

## 5 Conclusions

Our study demonstrates the value of historical DNA and natural history museum collections for investigating temporal patterns of genetic diversity. By combining historical temporal sampling with modern collections, we quantified changes in genetic diversity in two *Plethodon* salamander communities. These results underscore the complexity of population response to ongoing environmental change and highlight the need to continue preserving time series collections housed in natural history museums. We provide a robust framework for using natural history collections to better understand and address the genomic impacts of biodiversity loss in a rapidly changing world.

## Author Contributions

**Kyle A. O'Connell:** conceptualization (equal), formal analysis (lead), funding acquisition (lead), investigation (lead), methodology (equal), validation (lead), visualization (lead), writing – original draft (lead), writing – review and editing (equal). **Carly R. Muletz‐Wolz:** methodology (supporting), resources (supporting), writing – review and editing (supporting). **Addison Wynn:** conceptualization (supporting), methodology (supporting), resources (supporting), validation (supporting), writing – review and editing (supporting). **Kelly R. Zamudio:** methodology (supporting), writing – review and editing (supporting). **Karen R. Lips:** methodology (supporting), writing – review and editing (supporting). **Amy Ellison:** resources (supporting), writing – review and editing (supporting). **Rayna C. Bell:** conceptualization (supporting), funding acquisition (supporting), methodology (supporting), project administration (supporting), resources (supporting), supervision (lead), visualization (supporting), writing – original draft (supporting), writing – review and editing (equal).

## Funding

This work was supported by the National Science Foundation, IOS‐2131060 and the Global Genome Initiative Postdoctoral Funds.

## Ethics Statement

All animal sample collection was conducted under IACUC protocol 2018‐08. Sample collection was approved by Tennessee Wildlife Resources Agency (permit 1725), Virginia Department of Game and Inland Fisheries (permit 062344), North Carolina Wildlife Resources Commission (permit 18‐SC01260), with USDA Forest Service approval for the George Washington and Jefferson National Forests and USDA approval for National Forests in North Carolina (Appalachian Ranger District File Code 2720).

## Consent

The authors have nothing to report.

## Conflicts of Interest

The authors declare no conflicts of interest.

## Supporting information


Appendix S1.

Appendix S2.

## Data Availability

All codes, target‐capture probe resources, and input files needed to recreate the analyses herein can be found on Dryad at https://datadryad.org/dataset/doi:10.5061/dryad.76hdr7t86. Raw FASTQ files are available at NCBI Bioproject PRJNA692381, and accessions are in Table S1.
