# Supplementary material for: Using Serially Collected Specimens to Investigate the Potential Population Genetic Consequences of Reported Declines in Eastern Woodland Salamanders
Source: Ecol Evol. 2026 Jan 18;16(1):e72805. doi: 10.1002/ece3.72805 (PMC12812449; doi:10.1002/ece3.72805)
Supplement: Supplementary file 2 — Appendix S2. [file ECE3-16-e72805-s001.docx]

*Table S1: See attached Excel File showing full statistics for all samples used in this study.*

*Table S2: Summary of number of specimens used in downstream genomic analyses for each species / time point / site.*

| Species | Locality | 1960s–1970s | 1980s–1990s | 2018–2019 |
| --- | --- | --- | --- | --- |
| *P. cinereus* | IGG | 10 | 6 | – |
| *P. cinereus* | SG | 10 | – | – |
| *P. cylindraceus* | IGG | 5 | 3 | 4 |
| *P. glutinosus* | SG | 9 | 8 | 3 |
| *P. montanus* | IGG | 8 | – | 8 |
| *P. montanus* | SG | 10 | – | 8 |
| *P. welleri* | IGG | 9 | – | – |
| *P. yonahlossee* | IGG | 8 | 10 | 7 |
| *P. yonahlossee* | SG | 8 | 4 | 3 |

*Table S3: Summary statistics for each of the six datasets including three with all reads and three that were downsampled. For the all reads and the downsampled data, we included one dataset with SNPs from all captured sequences, one with only non-coding sites (flanking regions), and one with only coding sites pruned based on exonic positions from our probe sequences. We show here results for the all reads followed by the downsampled data for the total number of SNPs (SNPs), number of loci that passed filtering from which SNPs were called (Loci), the number of SNPs called for a given locus (SNPs / Locus), the percent of non-monomorphic loci (% Var. Loci), the mean depth per SNP (Mean Depth), the mean variable loci per species (Mean Vari.Loci / Species), and finally, the mean SNPs per species (Mean SNPs / Species). All datasets were filtered to sites with no more than 50% missing data and included 141 samples.*

| Sites | SNPs | | Loci | | SNPs / Locus | | % Var. Loci | | Mean Depth | | Mean Var. Loci / Species | | Mean SNPs / Species | |
| --- | --- | --- | --- | --- | --- | --- | --- | --- | --- | --- | --- | --- | --- | --- |
| Reads | all | down | all | down | all | down | all | down | all | down | all | down | all | down |
| all-sites | 31049 | 25111 | 2229 | 2091 | 13.9 | 12.0 | 86.7 | 81.3 | 57.1 | 32.2 | 249.4 | 231.9 | 4242.9 | 3534.3 |
| noncoding | 4203 | 2334 | 1295 | 888 | 3.2 | 2.6 | 50.3 | 34.5 | 31.3 | 20.0 | 130.7 | 91.3 | 474.0 | 258.2 |
| coding | 26846 | 2334 | 2222 | 2083 | 12.1 | 10.9 | 86.4 | 81.0 | 61.1 | 33.4 | 248.6 | 231.4 | 3769.0 | 3249.1 |

*Table S4: Results of ANOVA comparing genetic diversity between time points for each species and site. Statistics were corrected with Tukey post hoc corrections. Skull’s Gap (SG) and Indian Grave Gap (IGG).*

| Comparison1 | Comparison2 | Locality | lwr | upr | p | adj |
| --- | --- | --- | --- | --- | --- | --- |
| 1980’s – glutinosus | 1960’S – glutinosus | SG | 0.021058 | 0.018565 | 0.023551 | 0 |
| 2018 – glutinosus | 1960’S – glutinosus | SG | 0.026556 | 0.024063 | 0.029049 | 0 |
| 2018 – glutinosus | 1980’s – glutinosus | SG | 0.005498 | 0.003005 | 0.007991 | 0 |
| 2018 – montanus | 1960’S – montanus | SG | 0.006301 | 0.003808 | 0.008794 | 0 |
| 1980’s – yonahlossee | 1960’S – yonahlossee | SG | 0.002463 | 0.004956 | 0.000031 | 0.056461 |
| 2018 – yonahlossee | 1960’S – yonahlossee | SG | 0.008764 | 0.006271 | 0.011257 | 0 |
| 2018 – yonahlossee | 1980’s – yonahlossee | SG | 0.011226 | 0.008733 | 0.01372 | 0 |
| 1980’s – cinereus | 1960’S – cinereus | IGG | 0.001848 | 0.003514 | 0.000181 | 0.015420 |
| 1980’s – cylindraceus | 1960’S – cylindraceus | IGG | 0.009280 | 0.007613 | 0.010946 | 0 |
| 2018 – cylindraceus | 1960’S – cylindraceus | IGG | 0.001120 | 0.000546 | 0.002786 | 0.548183 |
| 2018 – cylindraceus | 1980’s – cylindraceus | IGG | 0.008159 | 0.009826 | 0.006493 | 0 |
| 2018 – montanus | 1960’S – montanus | IGG | 0.001869 | 0.000203 | 0.003535 | 0.013299 |
| 1980’s – yonahlossee | 1960’S – yonahlossee | IGG | 0.008452 | 0.006786 | 0.010119 | 0 |
| 2018 – yonahlossee | 1960’S – yonahlossee | IGG | 0.003264 | 0.001598 | 0.004931 | 0 |
| 2018 – yonahlossee | 1980’s – yonahlossee | IGG | 0.005187 | 0.006854 | 0.003521 | 0 |

| Comparison1 | Comparison2 | Locality | lwr | upr | p | adj |
| --- | --- | --- | --- | --- | --- | --- |
| 1980’s – glutinosus | 1960’S – glutinosus | SG | 0.021058 | 0.018565 | 0.023551 | 0 |
| 2018 – glutinosus | 1960’S – glutinosus | SG | 0.026556 | 0.024063 | 0.029049 | 0 |
| 2018 – glutinosus | 1980’s – glutinosus | SG | 0.005498 | 0.003005 | 0.007991 | 0 |
| 2018 – montanus | 1960’S – montanus | SG | 0.006301 | 0.003808 | 0.008794 | 0 |
| 1980’s – yonahlossee | 1960’S – yonahlossee | SG | 0.002463 | 0.004956 | 0.000031 | 0.056461 |
| 2018 – yonahlossee | 1960’S – yonahlossee | SG | 0.008764 | 0.006271 | 0.011257 | 0 |
| 2018 – yonahlossee | 1980’s – yonahlossee | SG | 0.011226 | 0.008733 | 0.01372 | 0 |
| 1980’s – cinereus | 1960’S – cinereus | IGG | 0.001848 | 0.003514 | 0.000181 | 0.015420 |
| 1980’s – cylindraceus | 1960’S – cylindraceus | IGG | 0.009280 | 0.007613 | 0.010946 | 0 |
| 2018 – cylindraceus | 1960’S – cylindraceus | IGG | 0.001120 | 0.000546 | 0.002786 | 0.548183 |
| 2018 – cylindraceus | 1980’s – cylindraceus | IGG | 0.008159 | 0.009826 | 0.006493 | 0 |
| 2018 – montanus | 1960’S – montanus | IGG | 0.001869 | 0.000203 | 0.003535 | 0.013299 |
| 1980’s – yonahlossee | 1960’S – yonahlossee | IGG | 0.008452 | 0.006786 | 0.010119 | 0 |
| 2018 – yonahlossee | 1960’S – yonahlossee | IGG | 0.003264 | 0.001598 | 0.004931 | 0 |
| 2018 – yonahlossee | 1980’s – yonahlossee | IGG | 0.005187 | 0.006854 | 0.003521 | 0 |

|  |  | |  | |  | |  | |  | |  | |  | |
| --- | --- | --- | --- | --- | --- | --- | --- | --- | --- | --- | --- | --- | --- | --- |
|  |  |  |  |  |  |  |  |  |  |  |  |  |  |  |
|  |  |  |  |  |  |  |  |  |  |  |  |  |  |  |
|  |  |  |  |  |  |  |  |  |  |  |  |  |  |  |
|  |  |  |  |  |  |  |  |  |  |  |  |  |  |  |

*
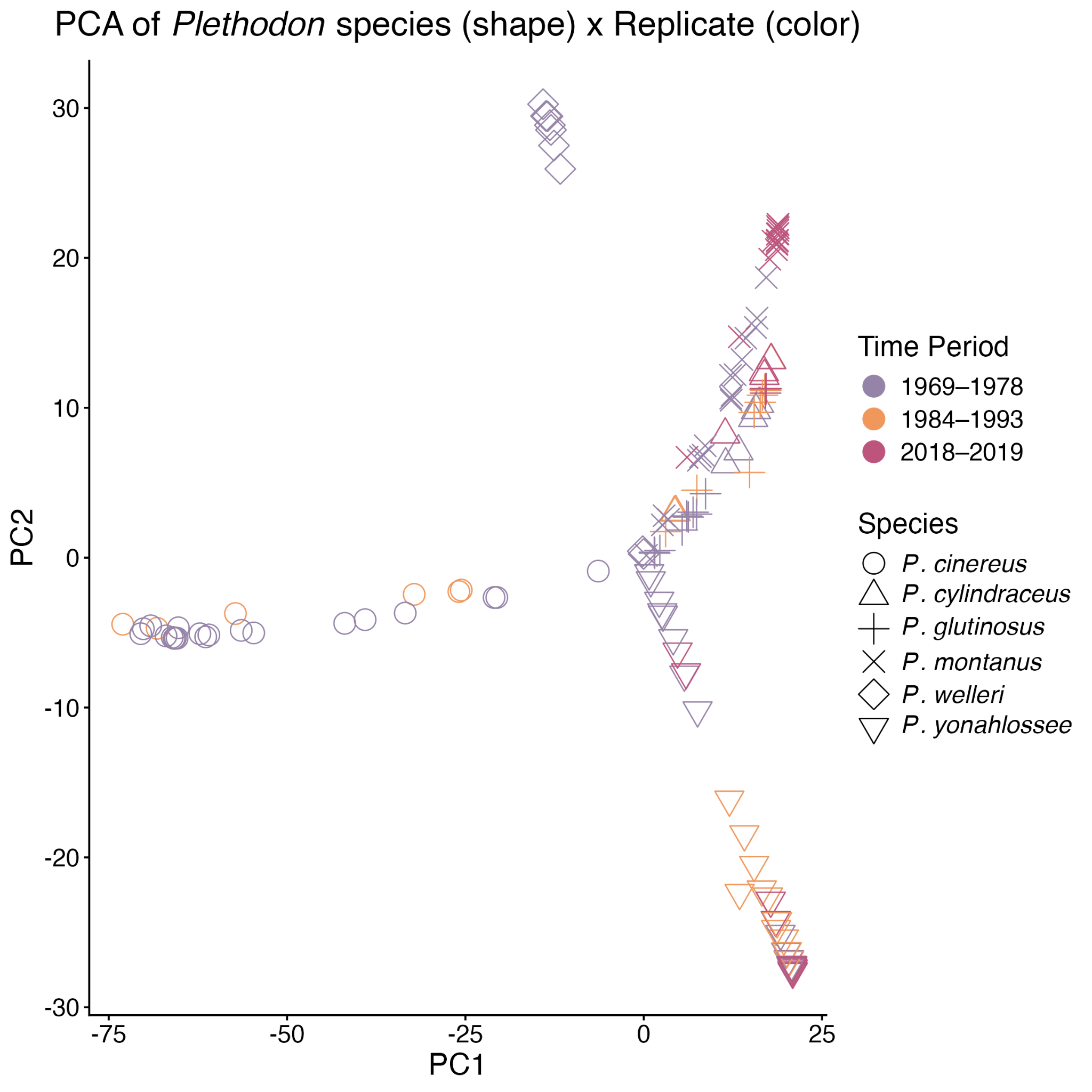
*

*Figure S1: Principal Components Analysis of all species and sample types showing that samples cluster by species rather than by tissue preservation type. Colors correspond to time period, with purple as historic liver samples (1960s–1970s), orange as frozen blood samples (1980s–1990s), and maroon as frozen liver (2018–2019).*

*
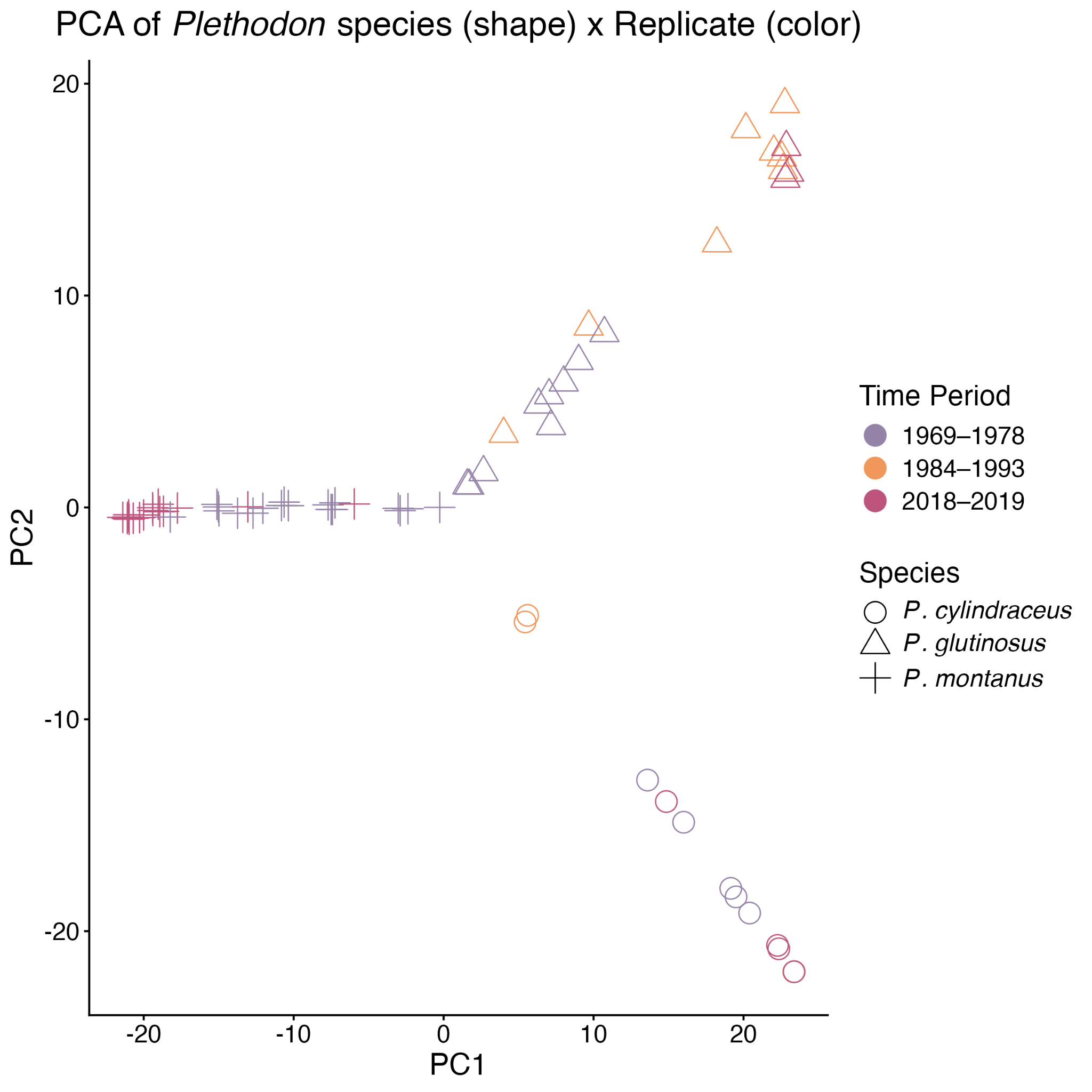
*

*Figure S2: Principal Components Analysis with the three most closely related species showing that samples cluster by species rather than by tissue preservation type.Colors correspond to time period, with purple as historic liver samples (1960s–1970s), orange as frozen blood samples (1980s–1990s), and maroon as frozen liver (2018–2019).*


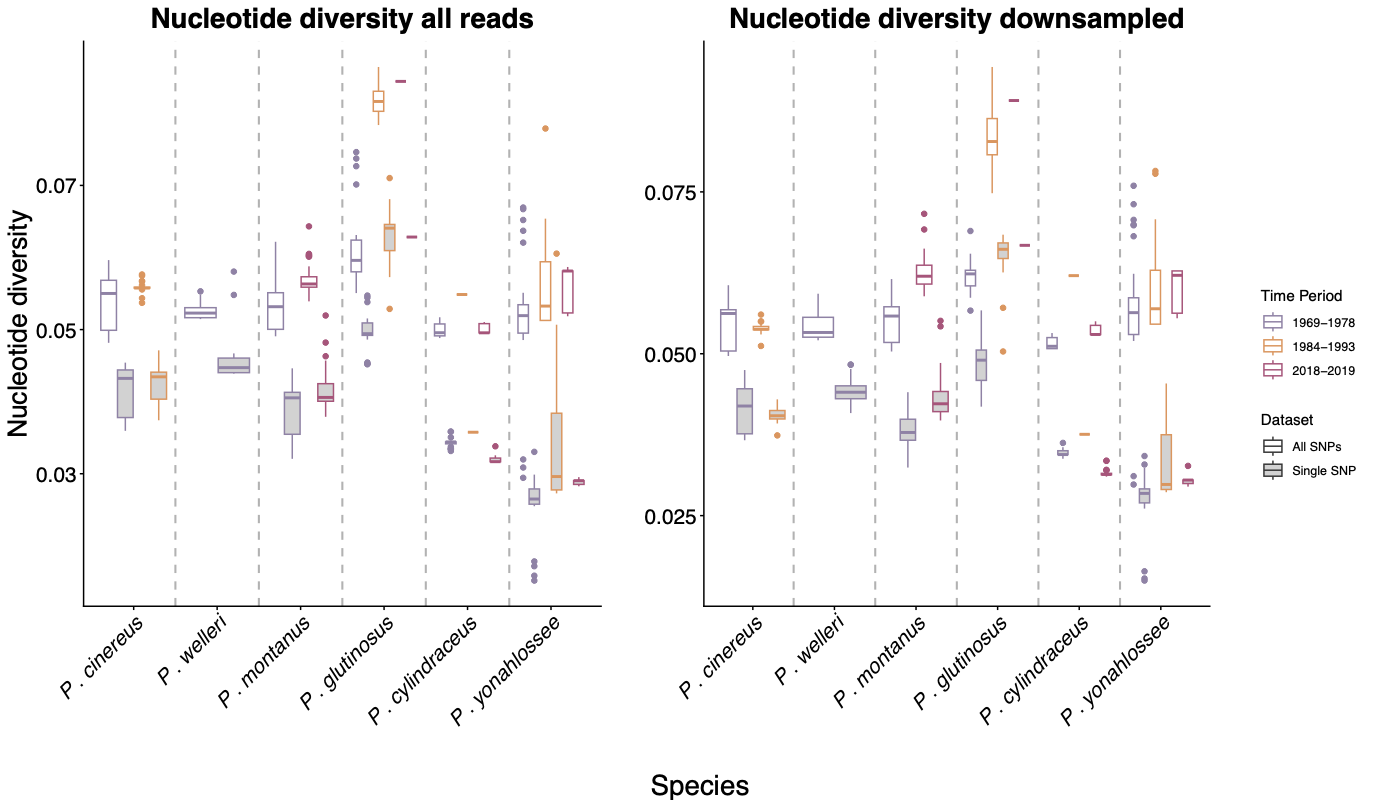


Figure S3: *Plot of nucleotide diversity comparing estimates derived from single SNP vs. all SNP datasets for the all reads and downsampled data for Skull’s Gap (SG) and Indian Grave Gap (IGG). Colors correspond to time period, with purple as historic liver samples (1960s–1970s), orange as frozen blood (1980s–1990s), and maroon as frozen liver (2018–2019). Results show that estimates of nucleotide diversity are biased downward when using a single SNP per locus.*

*
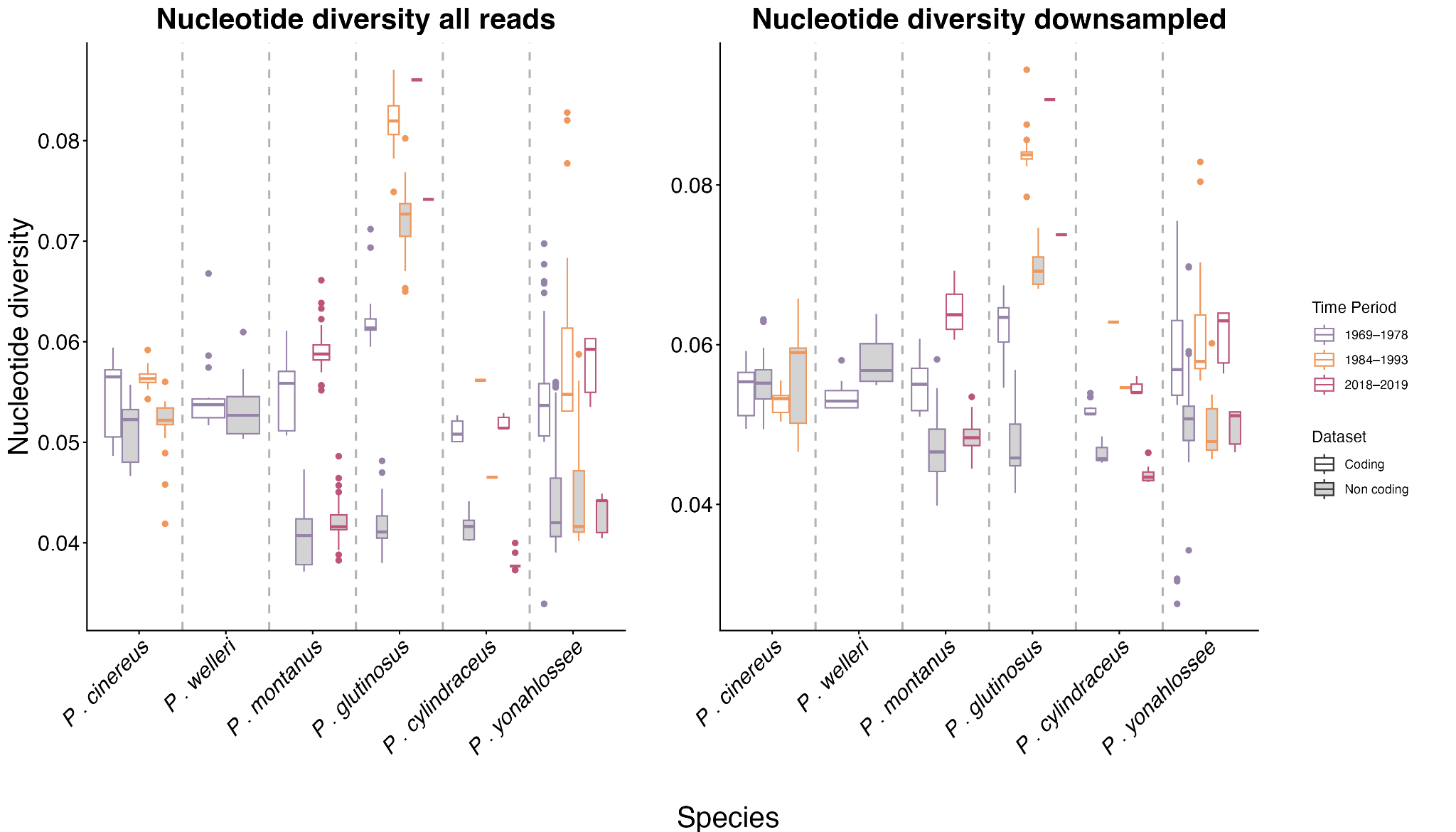
*

*Figure S4: Plot of nucleotide diversity comparing estimates derived from coding vs. non coding datasets for the all reads and downsampled data for Skull’s Gap (SG) and Indian Grave Gap (IGG). Colors correspond to time period, with purple as historic liver samples (1960s–1970s), orange as frozen blood (1980s–1990s), and maroon as frozen liver (2018–2019). Results show that estimates of nucleotide diversity are largely biased downward when using the downsampled data.*


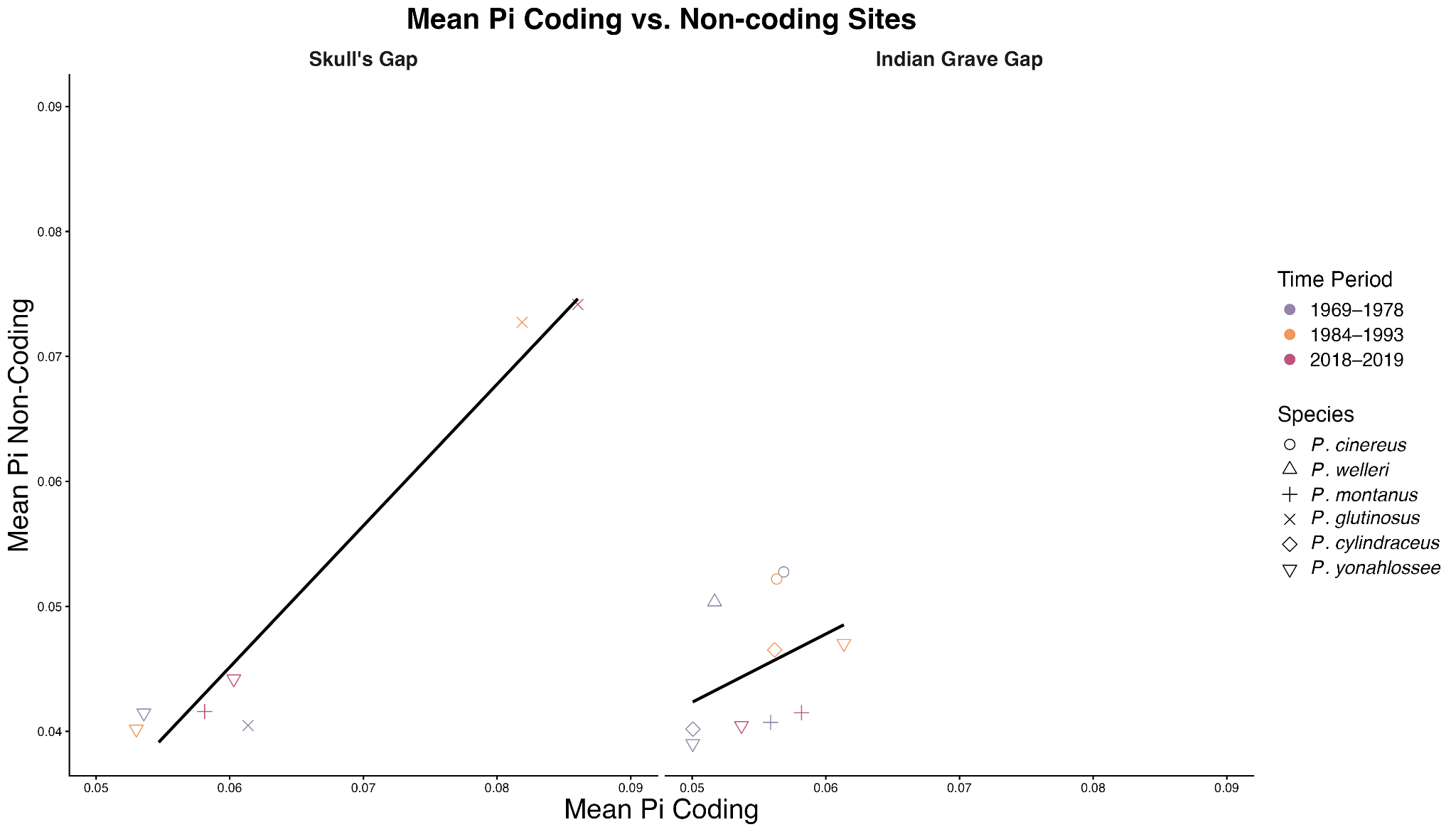


*Figure S5: Plot of genetic diversity estimates for coding vs. non-coding sites, showing a skew towards higher diversity estimates for coding sites compared with non-coding sites with Skull’s Gap (SG) on the left and Indian Grave Gap on the right.*

*
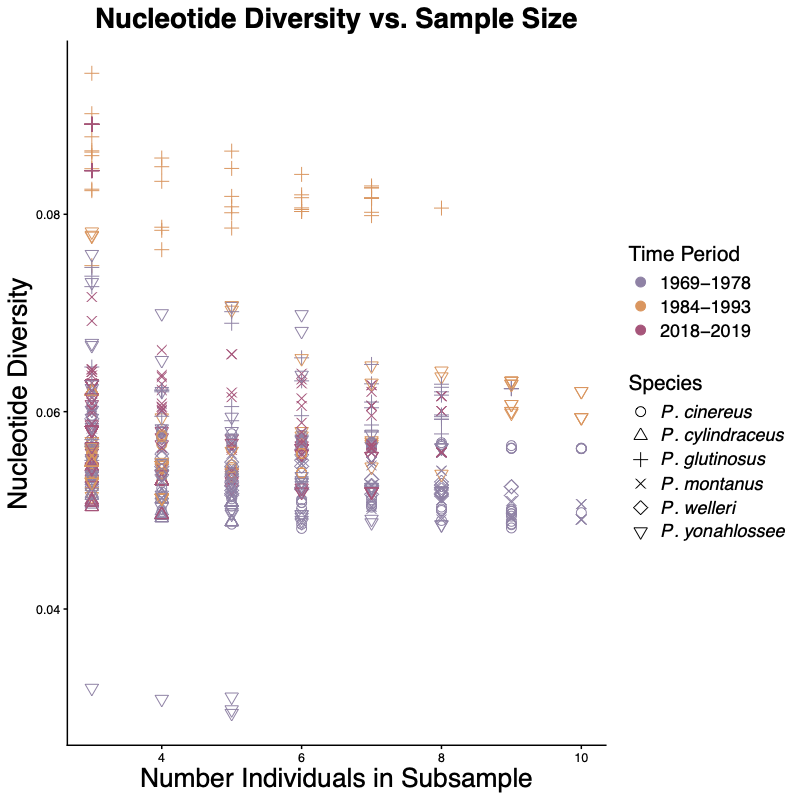
*

*Figure S6: Estimates of nucleotide diversity across permutations with varying numbers of individuals ranging from 3–10. We implemented permutation tests to control for varying sample sizes, results here show that variance in estimates declines as one adds samples to the estimate. Colors correspond to time period, with purple as historic liver samples (1960s–1970s), orange as frozen blood samples (1980s–1990s), and maroon as frozen liver (2018–2019).*

*
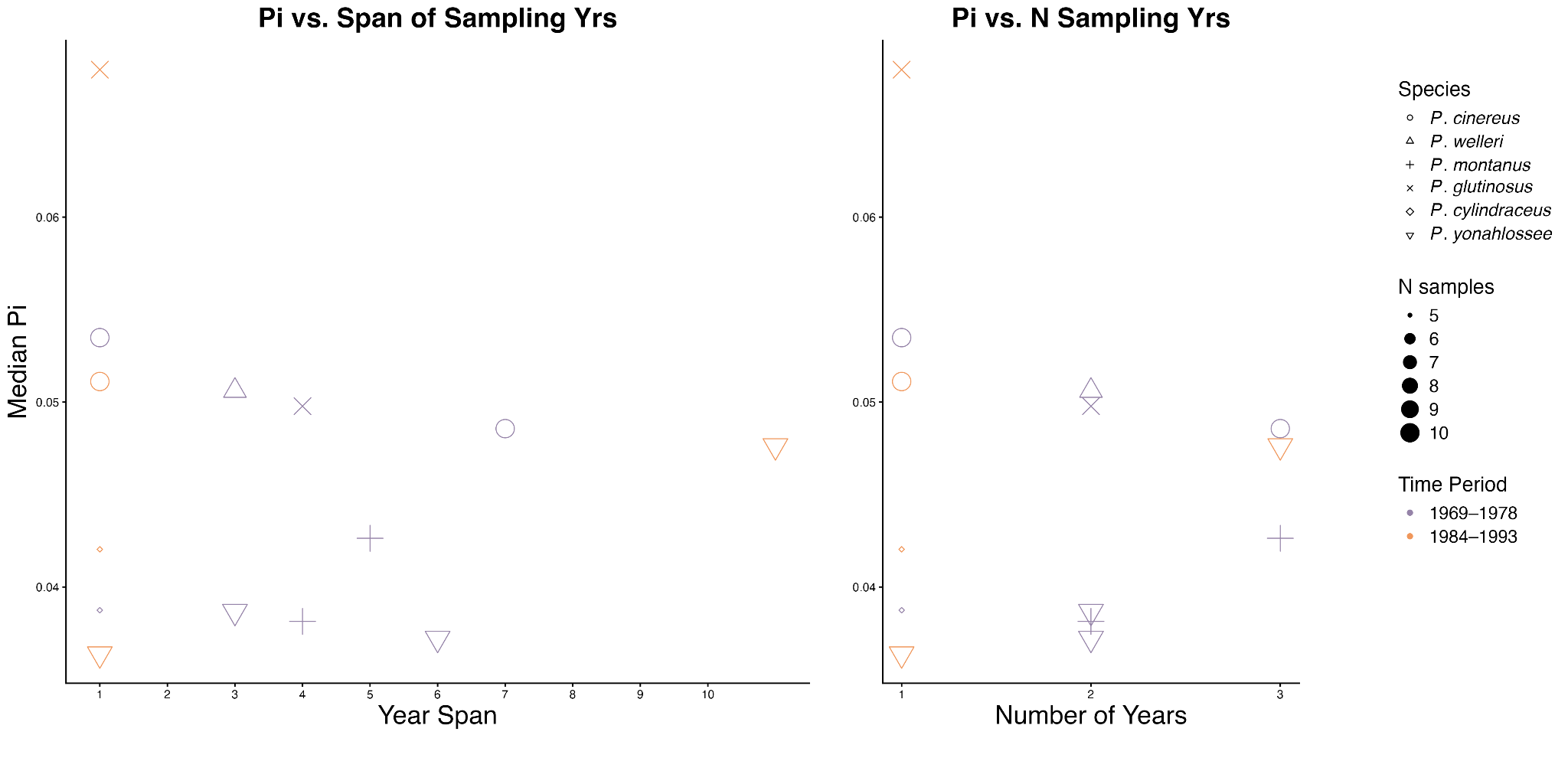
*

*Figure S7: Plot of median nucleotide diversity across permutations for each species and site for specimens collected by Richard Highton (historic liver and frozen blood). Left hand plot shows the span of years within a sample, calculated by the difference between the newest and oldest year for a given species + site. The plot on the right shows the number of years included which ranged from one to three. Colors correspond to time period, with purple as historic liver samples (1960s–1970s) and orange as frozen blood samples (1980s–1990s).*

*
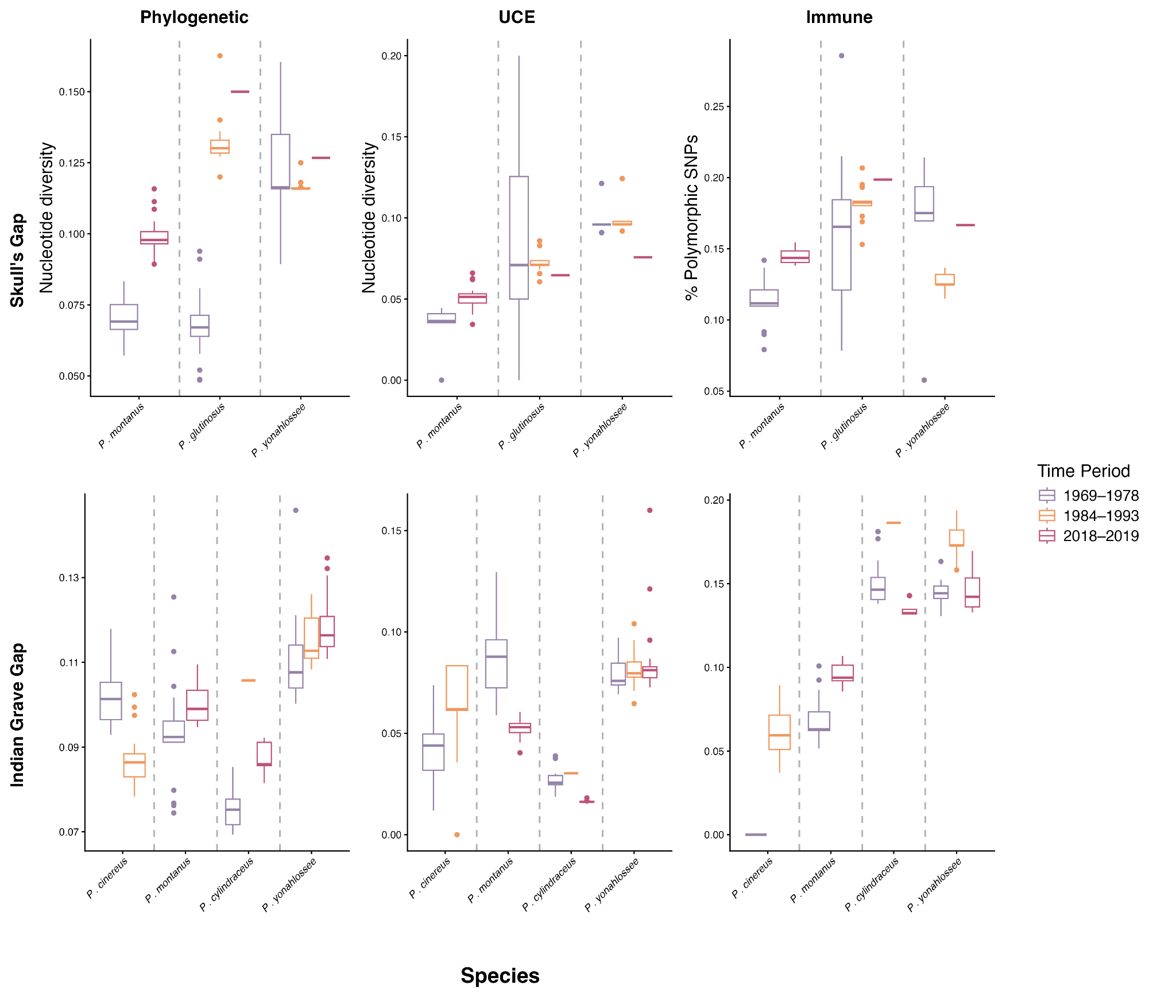
*

*Figure S8: Estimates of nucleotide diversity for different marker sets in populations with sequence data from at least two time points: (A) phylogenetic loci, (B) UCE loci, and (C) percent polymorphic SNPs for the three immune loci for Skull’s Gap (SG) and Indian Grave Gap (IGG). Colors correspond to time period, with purple as historic liver samples (1960s–1970s), orange as frozen blood samples (1980s–1990s), and maroon as frozen liver (2018–2019).*

*
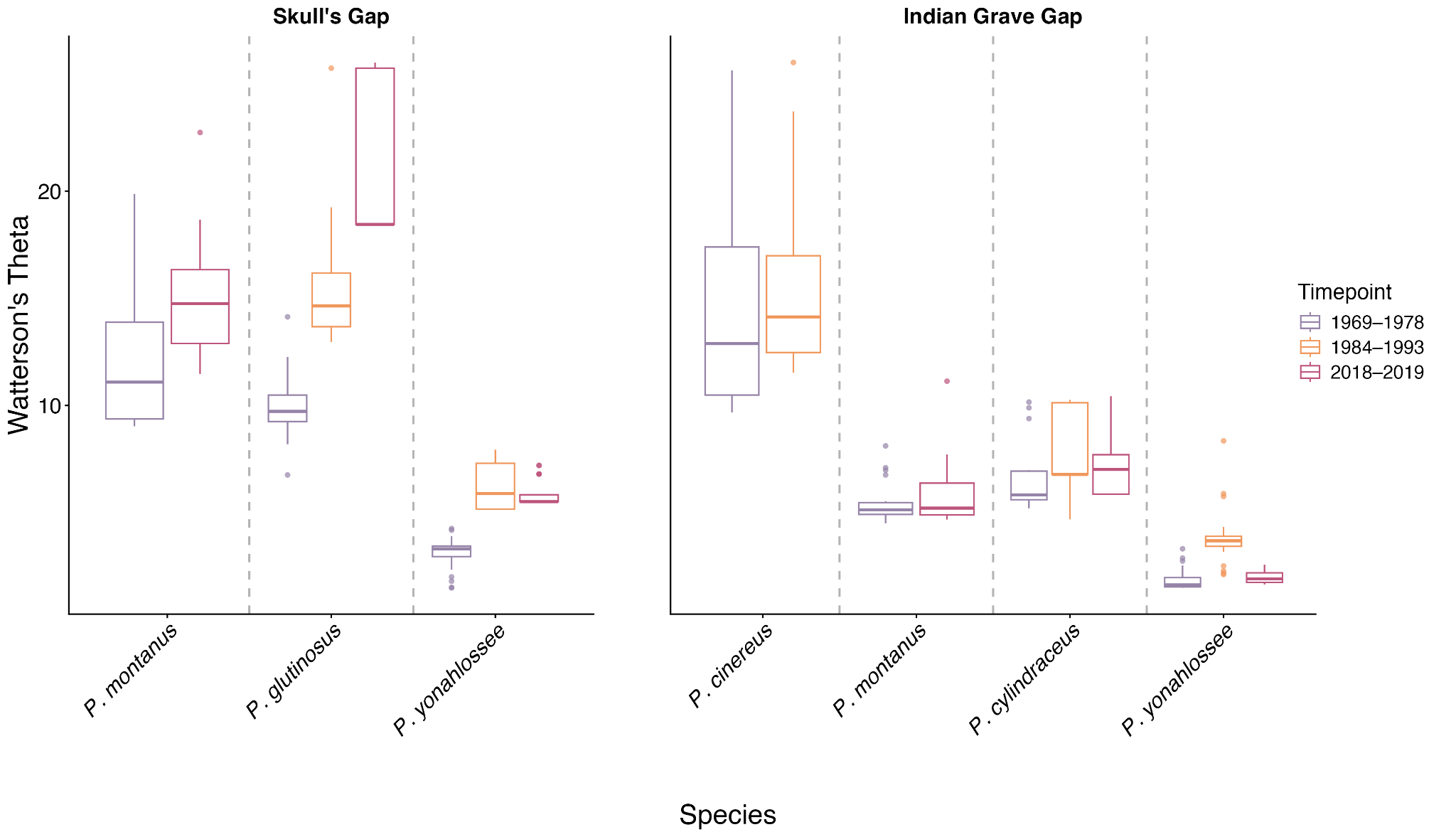
*

*Figure S9: Estimates of Watterson’s Theta for all species at Skull’s Gap (SG) and Indian Grave Gap (IGG). Colors correspond to time period, with purple as historic liver samples (1960s–1970s), orange as frozen blood (1980s–1990s), and maroon as frozen liver (2018–2019).*
